# Prevalence of Dietary Supplement Use by Athletes: Systematic Review and Meta-Analysis

**DOI:** 10.1007/s40279-015-0387-7

**Published:** 2015-10-06

**Authors:** Joseph J. Knapik, Ryan A. Steelman, Sally S. Hoedebecke, Krista G. Austin, Emily K. Farina, Harris R. Lieberman

**Affiliations:** US Army Research Institute of Environmental Medicine, 10 General Greene Ave, Building 42, Natick, MA 01760 USA; US Army Public Health Center, Aberdeen Proving Ground, Gunpowder, MD USA; Oak Ridge Institute for Education and Health, Belcamp, MD USA; Serenity Hill Nutrition, Street, MD USA

## Abstract

**Background:**

Dietary supplements (DSs) are commercially available products consumed as an addition to the usual diet and are frequently ingested by athletes.

**Objective:**

Our objective was to examine the prevalence of DS use by athletes.

**Data Sources:**

PubMed, Ovid MEDLINE, OVID Healthstar, and Cumulative Index to Nursing and Allied Health were searched for original research articles published up to August 2014. Search terms included specific sports, specific DSs, and other terms.

**Study Selection:**

Studies were selected if they were written in English, involved athletes, and provided a quantitative assessment of the proportion of athletes using specific DSs.

**Summary Measure:**

Percent of athletes using specific DSs.

**Synthesis of Data:**

Methodological quality of studies was assessed by three reviewers using an 8-point scale that included evaluations for sampling methods, sampling frame, sample size, measurement tools, bias, response rate, statistical presentation, and description of the participant sample. Where there were at least two investigations, meta-analysis was performed to obtain summary (pooled) prevalence estimates (SPEs) on (1) DS use prevalence by sport and sex, (2) DS use prevalence by elite versus non-elite athletic status, and (3) specific DS prevalence for all athletic groups combined. Meta-analyses included evaluations of homogeneity and publication bias.

**Results:**

A total of 159 unique studies met the review criteria. Methodological quality was generally low with an average ± standard deviation of 43 ± 16 % of available rating points. There was low homogeneity for SPEs when compiled by sport, athletic status, and/or specific DSs. Contributing to the lack of homogeneity were differences in studies’ objectives and types of assessments used (e.g., dietary surveys, interviews, questionnaires). Despite these limitations, the data generally indicated that elite athletes used DSs much more than their non-elite counterparts. For most DSs, use prevalence was similar for men and women except that a larger proportion of women used iron while a larger proportion of men used vitamin E, protein, and creatine. No consistent change in use over time was observed because even the earliest investigations showed relatively high use prevalence.

**Conclusion:**

It was difficult to generalize regarding DS use by athletes because of the lack of homogeneity among studies. Nonetheless, the data suggested that elite athletes used dietary supplements far more than their non-elite counterparts; use was similar for men and women with a few exceptions; use appeared to change little over time; and a larger proportion of athletes used DSs compared with the general US population. Improvements in study methodology should be considered in future studies especially (1) defining DSs for participants; (2) querying for very specific DSs; (3) using a variety of reporting timeframes (e.g., daily, 2–6 times/week, 1 time/week and <1 time/week); (4) reporting the sampling frame, number of individuals solicited, and number responding; (5) reporting characteristics of volunteers (and non-volunteers, if available); and (6) using similar methods on several occasions to examine possible temporal trends among athletes.

**Electronic supplementary material:**

The online version of this article (doi:10.1007/s40279-015-0387-7) contains supplementary material, which is available to authorized users.

## Key Points

**Table:** 

When dietary supplement use was compiled by sport, elite versus non-elite athletic status, and supplement type there was high variability in use prevalence among studies.
Elite athletes appeared to use dietary supplements much more than their non-elite counterparts.
For most dietary supplements, use prevalence appeared similar for men and women. Exceptions were that a larger proportion of women used iron and a larger proportion of men used vitamin E, protein, and creatine.

## Introduction

A dietary supplement is a commercially available product that is consumed as an addition to the usual diet and includes vitamins, minerals, herbs (botanicals), amino acids, and a variety of other products [1]. Marketing claims for some dietary substances include improvements in overall health status, enhancement of cognitive or physical performance, increase in energy, loss of excess weight, attenuation of pain, and other favorable effects. The Dietary Supplement Health and Education Act (DSHEA) of 1994 [2] established the regulatory framework for dietary supplements in the US. Since this act became law, US sales of dietary supplements has increased from $US4 billion in 1994 to $US33 billion in 2012 [3, 4], an eightfold increase over 18 years. Global sales of supplements were $US96 billion in 2012 and estimated at $US104 billion in 2013 [5].

Patterns of dietary supplement use may differ in distinctive subpopulations. Athletes in different sports may use different dietary supplements depending on the nature of the physical activities they perform and the desired outcomes from the dietary supplements. Athletes often perform intense and prolonged physical activity and often report that their primary reason for using dietary supplements is to enhance performance or recover from exercise [6–14], although improving/maintaining health can also be an important rationale [15–19]. In contrast, the general population appears to consume dietary supplements primarily for health-related reasons, with only minor interest in performance enhancement [20, 21]. Competitive athletes also need to be concerned with excessive use and possible adverse interactions due to polypharmacy [22, 23], and inadvertent doping due to the inadequate quality control of some dietary supplements [24, 25].

This paper presents a systematic literature review describing the prevalence of dietary supplement use in athletes. Where possible, meta-analyses were performed on dietary supplements by sport and sex, by elite versus non-elite athletic status, and by specific dietary supplements for all athletic groups combined. An older review on the prevalence of vitamin and mineral supplementation by athletes is also available [26].

## Methods

This investigation generally followed the Preferred Reporting Items for Systematic Reviews and Meta-Analyses (PRISMA) guidelines [27].

### Information Sources and Search

Literature searches were conducted in PubMed, Ovid MEDLINE (including OLDMEDLINE), OVID Healthstar, and Cumulative Index to Nursing and Allied Health Literature (CINAHL). No limitations were placed on the dates of the searches, and the final search was completed in August 2014. After reviewing PubMed medical subject headings for ‘dietary supplements’ and ‘athletes’, keywords selected for the search included athlete, sport, football, wrestling, soccer, ballet, dancing, running, gymnastics, swimming, basketball, hockey, tennis, softball, baseball, triathlete, triathlon, body building, weight lifting, volleyball, track, and crew. These keywords were combined with nutrition, dietary supplement, supplement, vitamin, mineral, amino acid, protein, herb, herbal, sport drink, sport bar, nutriceutical, neutraceuticals, food supplements, and food supplementation. To find additional studies, the reference lists of the articles obtained were searched, as was the literature database of an investigator with extensive experience with dietary supplement research.

### Eligibility Criteria

Articles were selected for the review if they were (1) written in English, (2) involved athletes, and (3) provided a quantitative assessment of the proportion of athletes using dietary supplements of any type, as defined by the DSHEA of 1994 [2]. Titles were first examined and abstracts were reviewed if the article appeared to involve athletes and either nutrition or dietary supplements. The full text of the article was retrieved if there was a possibility that dietary supplements were included within the investigation. Quantitative prevalence data could be contained within the text of the article, in tabular form, or presented in graphs. Data presented in graphic form were estimated. If the authors did not specifically reference dietary supplement prevalence, but data were available in the article to calculate it, then the article and the data were included in the review.

Not included in the review were studies that (1) examined athletic-related occupations (coaches, athletic trainers, physicians) or former athletes; (2) asked athletes about dietary supplements they would like to use (as opposed to actual use); (3) asked athletes about dietary supplement use they had observed; (4) mixed athletes with non-athletes, unless the athlete data were reported separately or could be calculated from the data provided; and (5) examined dietary supplement use during competitive events (because of the short timeframe and special, atypical circumstances). A number of studies that were identified did consider intake of dietary supplements in calculating nutritional intake of various athletic groups but did not specifically report the dietary supplement prevalence and so could not be used in this review. Abstracts, case studies, and case series were also not included. Stand-alone abstracts (without full-text articles) were excluded because they were difficult to locate, were generally not included in reference databases, and in many cases were not peer reviewed. Case studies and case series involved few individuals and were often published because they were atypical.

### Summary Measure

The summary statistic was the percent of athletes using a specific dietary supplement. Data extracted from each study were (1) the number of athletes using a particular dietary supplement and (2) the number of athletes in the entire sample. Dividing the former by the latter and multiplying by 100 % produced the use prevalence as a percent. Data from a number of studies required recalculation because authors expressed data as a percent of dietary supplement users rather than as a percent of the total sample.

### Considerations in Data Collection and Compilation

A “unique study” was defined as a single data collection period. Multiple publications could be produced from a single unique study. When there were multiple publications from a unique study, all dietary supplement prevalence reported in any of the publications were included in the data extraction process; however, the publication was included only once in the analyses so as not to bias prevalence estimates to studies with more publications.

To be included in the review, the dietary supplement examined in the publication had to be specifically identified. That is, dietary supplements grouped into categories like ‘antioxidant’, ‘pro-performance’, ‘herbal supplement’, ‘ergogenics’, ‘thermogenics’, ‘bodybuilding’, and the like were not included in the data extraction process. Exceptions were the general category of vitamins and minerals, which were included since so many studies reported these. Sport drinks, sports bars, and energy drinks were also included in the review for the same reason, although these substances are classified as ‘nutritional supplements’ since they contain nutritional labeling as specified by the DSHEA. Brand names that contained multiple supplements were not included unless the brand name could be identified as having a single major identifiable dietary supplement.

To summarize the raw data, two major tables were constructed, one describing the methodology used by each study [see Table S1 in the Electronic Supplementary Material (ESM)], the other containing the prevalences of the most frequently reported dietary supplements (see Table S2 in the ESM).

### Methodological Quality Ratings

Methodology quality of the investigations was assessed using the technique of Loney et al. [28], which was developed specifically for rating prevalence investigations. Studies were graded on an 8-point scale that included evaluations for sampling methods, sampling frame, sample size, measurement tools, bias, response rate, statistical presentation, and description of the participant sample. The eight items were rated as either ‘yes’ (1 point) or ‘no’ (no point), based on specific criteria. Thus, the maximum possible score was 8. Three authors independently rated each of the selected articles. Following the independent evaluations, the reviewers met to examine the scores and reconcile major differences. The average score of the reviewers served as the methodological quality score. Scores were converted to a percent of the total available points by dividing the average score for each study by 8 and multiplying by 100 %.

### Meta-Analyses

We used the Comprehensive Meta-Analysis Statistical Package, Version 3.2 (Biostat, Englewood, NJ, USA) to perform meta-analyses on (1) dietary supplement prevalence by sport and sex; (2) dietary supplement prevalence by elite versus non-elite athletic status; and (3) prevalence by specific dietary supplements for all athletic groups combined. These analyses required at least two studies and only studies that queried athletes about ‘current’, ‘usual’, or ‘regular’ use of dietary supplements were considered so that the reporting timeframe was similar. Studies asking about the use of dietary supplements at other times (e.g., last 2 months, last 6 months, etc.) were not included in the meta-analyses. For the sport-specific meta-analyses, the sport had to be explicit (e.g., football, basketball, tennis). If sports were grouped into broad categories (e.g., ‘combat sports’, ‘racquet sports’, ‘speed sports’, ‘power sports’, ‘endurance sports’, ‘ball sports’), they were not included. Elite athletes were defined as those competing professionally, at national or international level. Athletes competing at high schools, colleges, or universities were not considered elite. Only studies that involved adults and provided separate prevalence data for men and women were included in the elite versus non-elite meta-analyses. If sample sizes were not provided for a particular group or subgroup, the study could not be included because sample sizes are required for meta-analysis.

For all meta-analyses, we used a random model that considered users and non-users of dietary supplements to produce a summary prevalence estimate (SPE) and a summary 95 % confidence interval (S95 % CI) that represented the pooled data of all the individual investigations. Homogeneity of the pooled prevalence estimates was assessed using the *Q*- and the *I*^2^-statistics [29]. *I*^2^ indicated the percent of heterogeneity among studies, with smaller values denoting more homogeneity and larger values less homogeneity. In calculating *I*^2^, negative values were set to zero, which indicated very little heterogeneity [30]. Where there were four or more studies, publication bias was assessed by examining the symmetry of study data in funnel plots (1/standard error of prevalences vs. logit of the prevalences) and further appraising the information with the trim and fill evaluation. The trim and fill evaluation imputes the number of ‘missing’ studies in the funnel plot and re-computes an adjusted SPE and S95 % CI with the ‘missing’ studies included [31]. Where there were fewer than four studies, symmetry of studies about the funnel plot could not be interpreted.

## Results

**Fig. 1:**
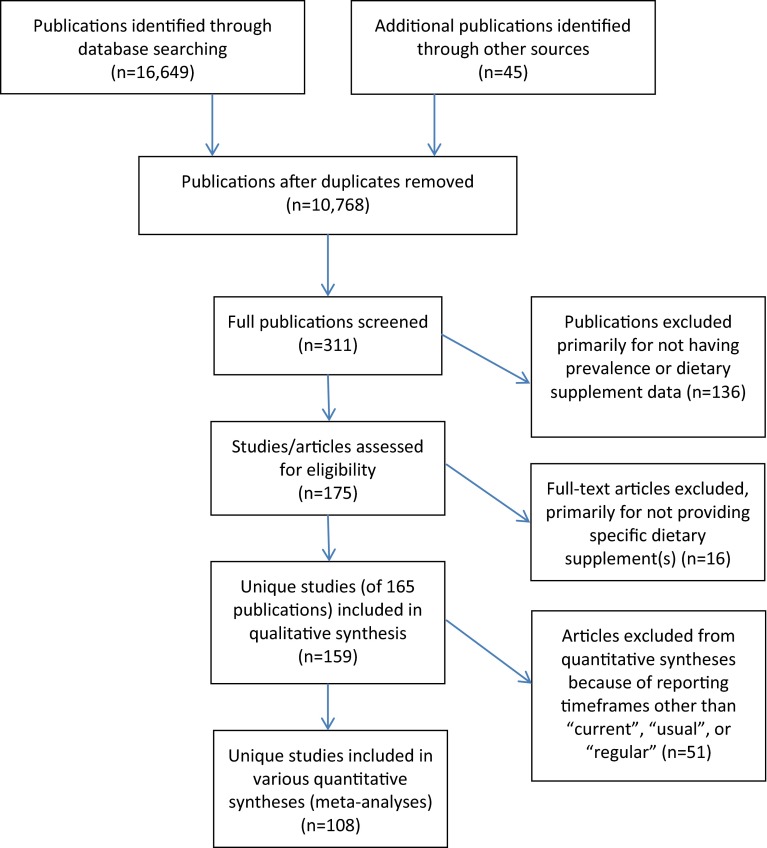
Publications included and excluded at each stage of literature review

Figure 1 shows the number of publications included and excluded at each stage of the literature search. The initial search identified 16,694 citations, 5926 of which were duplicate publications (from different databases) that were removed. Based on a review of titles and abstracts, 311 full articles were obtained for review, and subsequently 136 were removed for not having information on prevalence or meeting the exclusion criteria. A total of 175 studies were further reviewed, but 16 of these did not contain useful data because of the manner in which the dietary supplements were categorized (‘antioxidant’, ‘pro-performance’, ‘herbal supplement’, etc.). In total, 159 unique studies (and 165 published papers) finally met the inclusion criteria. Two unique studies produced two reports each [32–35], one resulted in three publications [36–38], and one resulted in four articles [12, 39–41].

### Study Methodologies

Table S1 in the ESM shows the participants and methods in the 159 unique investigations. In terms of specific sports, there were 14 studies of football players [7, 8, 42–53]; 12 of swimmers [7, 44, 45, 48, 54–61] and soccer players [7, 44, 45, 48, 52, 62–68]; ten of body builders [6, 69–77] and basketball players [7, 42–46, 52, 78–80]; nine of volleyball players [7, 43–47, 49, 52, 81]; eight of wrestlers [7, 42–45, 47, 48, 82] and tennis players [7, 43–48, 52]; seven of ballet dancers [83–89] and gymnasts [7, 44, 45, 90–93]; six with marathoners [36, 63, 94–97], baseball players [7, 43, 45, 46, 48, 52], triathletes [63, 98–102], and golfers [7, 44–47, 52]; five with dancers [47, 89, 103–105]; four with ultra-marathoners [106–109]; three with runners [110–112], figure skaters [15, 113, 114], alpine skiers [44, 48, 115], cheerleaders [7, 45, 48], cross-country runners [7, 44, 52], lacrosse players [44, 45, 48], softball players [7, 43, 52], and ice hockey players [44, 116, 117]; two with field hockey players [45, 48]; and one each with aerobic dancers [118], cyclists [119], divers [45], fencers [45], Nordic skiers [115], rugby players [120], sailors [121], speed skaters [122], synchronized swimmers [89], surfers [123], table tennis players [124], and weight lifters [63]. Eight studies involved athletes in ‘track and field’ sports without defining the specific sport [7, 16, 43, 44, 46, 104, 125, 126]. Other studies looked at groups of sports with elite athletes [9, 13, 14, 18, 19, 34, 35, 127–138] (including Olympic athletes [32, 139–145]), university or college athletes [11, 146–156], high school athletes [157–162], fitness/health/wellness club or gym users [10, 17, 95, 163–169], and other groups of athletes [12, 23, 79, 170–177].

The data in Table S1 in the ESM indicate that 63 of 159 unique studies (40 %) used questionnaires to obtain dietary supplement information from participants, and these were either focused on general or more specific (e.g., creatine or protein) dietary supplement. A total of 63 studies (40 %) centered on topics other than dietary supplements but included some items on general or specific dietary supplement use. Some studies used dietary records, asking athletes to record what they consumed in periods of 1 day (*n* = 1), 3 days (*n* = 26), 4 days (*n* = 2), 7 days (*n* = 10), or 14 days (*n* = 1), with some studies collecting data over several periods (*n* = 6). A few investigations used dietary recall (*n* = 7) and/or food frequency questionnaires (*n* = 4). Some studies interviewed athletes (*n* = 7) or used interviews in combination with other methods (*n* = 3). In a few cases (*n* = 3), the methods for collecting the study data were not stated.

The data in Table S1 of the ESM indicate that the athletes’ reporting timeframe differed among studies. There were 108 studies (68 %) that involved current, regular, or usual use of dietary supplements. Some studies focused on dietary supplement use in specific periods of time including the last 3 days (*n* = 1), last month (*n* = 5), last 2 months (*n* = 1), last 3 months (*n* = 3), last 6 months (*n* = 6), last 12 months (*n* = 5), in the bulking phase of body building (*n* = 1), for >6 months in the previous 3 years (*n* = 1), or use in athletes’ college sports careers (*n* = 1). A number of studies asked about dietary supplement use at any time, currently or in the past (*n* = 14). In some studies, the reporting timeframe was not clear (*n* = 13).

Table S1 in the ESM shows that the proportion of solicited athletes participating in the investigations ranged from 7 to 100 %, with 36 investigations (23 %) achieving ≥66 % participation and 32 studies (20 %) with participation of <66 %. In 89 studies (56 %), the proportion of the solicited athletes participating in the study was not specified. In two cases (1 %), the nature of the study was such that participation could not be determined (i.e., a case-control study and a study that only reported the proportion of high schools participating).

As shown in Table S1 in the ESM, rating from the methodological quality reviews ranged from 8 to 83 % of available points, with an average ± standard deviation rating of 43 ± 16 %. Only 54 studies (34 %) had scores of ≥50 % and only eight studies (5 %) had scores of ≥70 %. Very few investigations drew true random samples or specified a sampling frame. In some cases, lower methodological quality was associated with the fact that the study was not focused on dietary supplement prevalence. For example, studies on medication use or ‘doping’ [23, 135, 142, 143, 176] and studies on the nutritional intake [54, 62, 81, 133] included information on dietary supplement prevalence, but this was not the main purpose of the investigation. Table 1 shows that the methodological quality scores in the studies published in the 1969–1989 period were slightly lower than those published later, but the overall difference between the two publication periods (1969–1989 vs. 1990–2014) was only 6 %.

**Table 1:** Methodological quality scores at various publication times

Publication date (year grouping)	Studies (*n*)	Methodological quality score (mean ± SD)	One way analysis of variance *p* value
1969–1989	38	37 ± 15	0.04
1990–1999	32	45 ± 14	
2000–2009	50	43 ± 13	
2010–2014	39	46 ± 16	

*SD* standard deviation

### Dietary Supplement Use Reported in Individual Investigations

Table S2 in the ESM shows the prevalence of dietary supplement use documented in each study. Information on the prevalence of any dietary supplement use was included in 95 studies (60 %). The use of any vitamins and/or minerals was reported in 67 investigations (42 %), multivitamin/multimineral use in 67 studies (42 %), and specific vitamins and/or minerals in 70 studies (44 %). Amino acid and/or protein supplementation was reported in 82 investigations (52 %), while creatine use was reported in 50 (31 %). There were 27 studies (17 %) that reported on specific herbal supplements. Sports drink use was reported in 33 studies (21 %), sports bar use in 18 studies (11 %), substances containing omega-3 fatty acids in 11 studies (7 %), caffeine use in eight studies (5 %), and energy drink consumption in seven studies (4 %).

### Meta-Analyses on Dietary Supplement Prevalence

Table 2 presents the meta-analyses conducted on studies that provided data by sport, sex, and specific dietary supplements and where there were at least two investigations involving specific dietary supplements. Very few investigations provided the necessary data for conducting meta-analyses by these categories. Some studies that would have been appropriate for these analyses did not provide sample sizes by sport [43–45, 47, 48, 52], while other studies that did provide sport and sample size did not separate dietary supplement data by sex [56, 58, 60, 61, 96, 106, 109, 112, 113, 118, 121]. Where appropriate data were available, many of the SPEs had wide S95 % CIs and low homogeneity. Of the 31 analyses in Table 2, only about half (*n* = 16) had *Q* statistic *p* values >0.05, and 29 % (*n* = 9) had *I*^2^ values ≤50 %. Despite this, the data suggested that the largest prevalence of dietary supplement use was among male soccer players and body builders, with other groups having lower use prevalences.

**Table 2:** Summary data on prevalence of dietary supplement use of athletes by sport and sex

Sport	Subgroup	Dietary supplement	Studies	Nationality	Individual study prevalence (%)	Total sample size (*n*)	SPE [% (S95 % CI)]	Homogeneity of SPE	
								*Q* statistic *p* value	*I* ^2^ (%)
Football	College men	Any vitamin	Jonnalagadda 2001 [50]	US	23				
			Cole et al. 2005 [51]	US	4	59	11 (2–48)	0.06	71
		Creatine	Greenwood et al. 2000 [46]	US	67				
			Jonnalagadda et al. 2001 [50]	US	36	107	52 (23–80)	<0.01	88
	High school males	Creatine	Swirzinski 2000 [8]	US	29				
			Scofield and Unruh 2006 [52]	US	26	182	29 (23–36)	0.77	0
		Any vitamin or mineral	Sobal 1994 [44]	US	39				
			Scofield and Unruh 2006 [52]	US	17	95	31 (14–56)	0.16	50
Soccer	Elite men	Any DS	Tscholl et al. 2008 [66]	Mixed	43				
			Aljaloud and Ibrahim 2013 [68]	Saudi Arabian	93	1577	76 (15–98)	<0.01	98
		Any vitamin	Burke et al. 1991 [63]	Australian	14				
			Waddington et al. 2005 [65]	UK	58	2234	39 (26–55)	<0.01	95
			Tscholl et al. 2008 [66]	Mixed	42				
		Amino acids	Tscholl et al. 2008 [66]	Mixed	8				
			Aljaloud and Ibrahim 2013 [68]	Saudi Arabian	25	841	14 (4–38)	<0.01	96
		Creatine	Waddington et al. 2005 [65]	UK	37				
			Tscholl et al. 2008 [66]	Mixed	8	2283	17 (5–47)	<0.01	99
			Aljaloud and Ibrahim 2013 [68]	Saudi Arabian	15				
Body-building	Men	Any DS	Faber 1987 [69]	South African	63				
			Linseisen et al. 1993 [74]	German	100	337	82 (57–94)	<0.01	90
			Karimian et al. 2011 [77]	Iranian	87				
		Any vitamin	Brill and Keane 1994 [6]	US	70				
			Karimian et al. 2011 [77]	Iranian	52	460	61 (43–77)	<0.01	93
		Any mineral	Brill and Keane 1994 [6]	US	49				
			Karimian et al. 2011 [77]	Iranian	15	460	29 (7–68)	<0.01	98
		MVM	Faber 1987 [69]	South African	37				
			Sandoval et al. 1989 [70]	US	20	128	42 (29–55)	0.18	42
			Andersen et al. 1995 [76]	US	51				
		Vit A	Faber 1987 [69]	South African	10				
			Karimian et al. 2011 [77]	Iranian	11	324	11 (8–15)	0.68	0
		Vit B or B complex	Faber 1987 [69]	South African	14				
			Andersen et al. 1995 [76]	Iranian	28	123	20 (9–39)	0.04	76
		Vit C	Faber 1987 [69]	South African	31				
			Andersen et al. 1995 [76]	Iranian	46	123	38 (24–55)	0.08	68
		Protein	Faber 1987 [69]	South African	59				
			Linseisen et al. 1993 [74]	German	100	346	63 (44–79)	<0.01	83
			Brill and Keane 1994 [6]	US	61				
			Andersen et al. 1995 [76]	US	38				
		Amino acids	Brill and Keane 1994 [6]	US	51				
			Andersen et al. 1995 [76]	US	58	259	52 (46–58)	0.44	0
	Women	Any DS	Walberg-Rankin et al. 1993 [75]	US	100				
			Karimian et al. 2011 [77]	Iranian	11	256	50 (1–99)	<0.01	89
		Any vitamin	Brill and Keane 1994 [6]	US	76				
			Karimian et al. 2011 [77]	Iranian	4	349	27 (1–96)	<0.01	99
		Any mineral	Brill and Keane 1994 [6]	US	57				
			Karimian et al. 2011 [77]	Iranian	10	349	28 (3–81)	<0.01	99
		MVM	Sandoval et al. 1989 [70]	US	50				
			Walberg-Rankin et al. 1993 [75]	US	33	12	42 (18–70)	0.56	0
		Iron	Walberg-Rankin et al. 1993 [75]	US	17				
			Karimian et al. 2011 [77]	Iranian	5	256	6 (3–15)	0.25	23
		Calcium	Walberg-Rankin et al. 1993 [75]	US	17				
			Karimian et al. 2011 [77]	Iranian	7	256	8 (5–12)	0.4	0
		Protein	Walberg-Rankin et al. 1993 [75]	US	50				
			Brill and Keane 1994 [6]	US	54	105	53 (44–63)	0.87	0
		Amino acids	Walberg-Rankin et al. 1993 [75]	US	100				
			Brill and Keane 1994 [6]	Iranian	59	105	73 (27–95)	0.13	55
Ballet	Elite women	Any DS	Calabrese et al. 1983 [84]	US	40				
			Zenic et al. 2010 [89]	Croatian	76	46	59 (23–87)	0.02	83
Triathlon	Men	Any DS	Burke et al. 1991 [63]	Australian	44				
			Knez and Peake 2010 [101]	Mixed	58	256	54 (48–60)	0.53	0
			Dolan et al. 2011 [102]	US	55				
	Women	Any DS	Knez and Peake 2010 [101]	Mixed	69				
			Dolan et al. 2011 [102]	US	53	207	56 (44–67)	0.27	19
Dance	Women	Any DS	Evers 1987 [103]	US	48				
			Sekulic et al. 2008 [105]	Serbian	24	67	30 (19–49)	0.11	55
			Zenic et al. 2010 [89]	Croatian	18				
		Sport drink	Sekulic et al. 2008 [105]	Serbian	24				
			Zenic et al. 2010 [89]	Croatian	18	46	22 (12–36)	0.1	76
Ultra- marathon	Men	Magnesium	Peters and Goetzsche 1997 [107]	Mixed	70				
			Knechtle et al. 2008 [108]	Mixed	45	170	60 (35–81)	0.03	79

*CI* confidence interval, *DS* dietary supplement, *MVM* multivitamins/multiminerals, *SPE* summary prevalence estimate, *S95* *% CI* summary 95 % CI, *vit* vitamin

Table 3 shows the results of the meta-analyses of selected dietary supplements (any dietary supplements, multivitamins/multiminerals, and vitamin C) comparing sex-specific elite and non-elite athletic groups. A wide variety of sports were represented in these comparisons. With few exceptions, the SPEs demonstrated low homogeneity, with only 4 of the 12 analyses (33 %) having *Q* statistic *p* values >0.05 and *I*^2^ values ≤50 %. There was generally little indication of publication bias, with the exception of any dietary supplement use in elite athletes. For elite athletes, the trim and fill adjusted SPE for any dietary supplement use was lower than the unadjusted values, but still higher than that of the non-elite athletes. Compared with non-elite athletes, a larger proportion of elite athletes also used multivitamins/multiminerals and vitamin C.

**Table 3:** Use of selected dietary supplement by elite and non-elite athletic status

Dietary supplement	Status	Sex	Studies	Nationality	Individual study prevalence (%)	Total sample size (*n*)	SPE [% (S95 % CI)]	Homogeneity of SPE		Trim and fill evaluation	
								*Q* statistic *p* value	*I* ^2^ (%)	Imputed studies (*n*)	Adjusted SPE [% (S95 % CI)]
Any DS	Elite	Men	Houston 1980 [54]	Canadian	75	2297	69 (60–78)	<0.01	94	6	55 (45–65)
			Snyder et al. 1989 [122]	US	60						
			Kleiner et al. 1990 [73]	US	90						
			Berglund 2001 [141]	Swedish	28						
			Sundgot-Borgen et al. 2003 [9]	Norwegian	51						
			Ziegler et al. 2003 [15]	US	65						
			Nieper 2005 [16]	UK	55						
			Huang et al. 2006 [198]	Canadian	71						
			Dascombe et al. 2010 [18]	Australian	89						
			Kondric et al. 2010 [124]	Slovenian	94						
			Kim et al. 2010 [13]	Korean	79						
			Kim et al. 2011 [144]	Korean	79						
			Lazic et al. 2011 [23]	Serbian	61						
		Women	Houston 1980 [54]	Canadian	50	1634	71 (62–79)	<0.01	90	6	60 (50–69)
			Snyder et al. 1989 [122]	US	86						
			Kleiner et al. 1990 [73]	US	100						
			Berglund 2001 [141]	Swedish	40						
			Sundgot-Borgen et al. 2003 [9]	Norwegian	54						
			Ziegler et al. 2003 [15]	US	76						
			Nieper et al. 2005 [16]	UK	75						
			Huang et al. 2006 [198]	Canadian	73						
			Dascombe et al. 2010 [18]	Australian	86						
			Kondric et al. 2010 [124]	Slovenian	83						
			Kim et al. 2010 [13]	Korean	82						
			Kim et al. 2011 [144]	Korean	82						
			Lazic et al. 2011 [23]	Serbian	61						
	Non elite	Men	Nowak et al. 1988 [78]	US	6	952	48 (27–70)	<0.01	97	0	48 (27–70)
			Neiman et al. 1989 [97]	US	30						
			Peters and Goetzsche 1997 [107]	South African	75						
			Sekulic et al. 2008 [105]	Serbian	14						
			Knez and Peake 2010 [101]	Mixed	58						
			Karimian et al. 2011 [77]	Iranian	87						
			Dolan et al. 2011 [102]	US	55						
		Women	Nowak et al. 1988 [78]	US	50	561	42 (22–66)	<0.01	94	0	42 (22–66)
			Neiman et al. 1989 [97]	US	27						
			Peters and Goetzsche 1997 [107]	South African	83						
			Sekulic et al. 2008 [105]	Serbian	24						
			Knez and Peake 2010 [101]	Mixed	69						
			Karimian et al. 2011 [77]	Iranian	11						
			Dolan et al. 2011 [102]	US	53						
MVM	Elite	Men	Houston 1980 [54]	Canadian	50	218	56 (50–63)	0.91	0	0	56 (50–63)
			Rosen et al. 1999 [115]	Norwegian	57						
			Ziegler et al. 2003 [15]	US	61						
			Kim et al. 2011 [144]	Korean	56						
		Women	Houston 1980 [54]	Canadian	42	192	58 (31–81)	<0.01	90	0	58 (31–81)
			Rosen et al. 1999 [115]	Norwegian	26						
			Ziegler et al. 2003 [15]	US	83						
			Kim et al. 2011 [144]	Korean	72						
	Non elite	Men	Sandoval et al. 1989 [70]	US	20	1397	33 (26–41)	<0.01	87	0	33 (26–41)
			Nieman et al. 1989 [97]	US	22						
			Worme et al. 1990 [99]	US	30						
			Krumbach et al. 1999 [7]	US	41						
			Froiland et al. 2004 [11]	US	22						
			Kristiansen et al. 2005 [151]	Canadian	52						
			Kim et al. 2011 [153]	Korean	30						
			Dolan et al. 2011 [102]	US	40						
		Women	Sandoval et al. 1989 [70]	US	50	735	39 (30–49)	<0.01	83	2	34 (24–44)
			Nieman et al. 1989 [97]	US	20						
			Worme et al. 1990 [99]	US	57						
			Krumbach et al. 1999 [7]	US	43						
			Froiland et al. 2004 [11]	US	26						
			Kristiansen et al. 2005 [151]	Canadian	63						
			Kim et al. 2011 [153]	Korean	30						
			Dolan et al. 2011 [102]	US	39						
Vitamin C	Elite	Men	Houston 1980 [54]	Canadian	38	44	36 (26–48)	0.94	0	^a^	^a^
			Rosen et al. 1999 [115]	Norwegian	37						
		Women	Houston 1980 [54]	Canadian	25	43	30 (23–40)	0.67	0	^a^	^a^
			Rosen et al. 1999 [115]	Norwegian	31						
	Non elite	Men	Neiman et al. 1989 [97]	US	14	1015	17 (14–20)	0.22	32	0	17 (14–20)
			Krumbach et al. 1999 [7]	US	20						
			Froiland et al. 2004 [11]	US	18						
			Kim et al. 2011 [153]	Korean	15						
		Women	Neiman et al. 1989 [97]	US	7	425	17 (12–25)	0.03	67	1	20 (13–29)
			Krumbach et al. 1999 [7]	US	24						
			Froiland et al. 2004 [11]	US	14						
			Kim et al. 2011 [153]	Korean	21						

*CI* confidence interval, *DS* dietary supplement, *MVM* multivitamin/multimineral, *SPE* summary prevalence estimate, *S95* *% CI* summary 95 % CI

^a^Not calculated with fewer than four studies

An attempt was made to examine dietary supplement use in all athletic groups combined; the results are shown in Table 4. There was very little homogeneity among the studies. Of the 61 analyses, only two had a *Q* statistic *p* value >0.05 and only one had an *I*^2^ ≤ 50 %. Despite this, the SPEs seem to indicate that the overall prevalence of dietary supplement use was high, with about 60 % of athletes using dietary supplements of any type and with vitamin/minerals, multivitamins/multiminerals, vitamin C, protein, sport drinks, and sport bars among the most commonly used. Male and female athletes had similar SPEs for most dietary supplements, with a few exceptions: compared with men, a larger proportion of women used iron and a smaller proportion used vitamin E, protein, and creatine. There was some indication of publication bias but these cases had little effect on the trim and fill adjusted SPEs.

**Table 4:** Prevalence of dietary supplement use in combined athletic groups

Dietary supplement	Sex	Studies (*n*)	SPE [% (S95 % CI)]	Homogeneity of SPE		Trim and fill evaluation	
				*Q* statistic *p* value	*I* ^2^	Imputed studies (*n*)	Adjusted SPE [% (S95 % CI)]
Any DS	M and F	61	60 (55–64)	<0.01	97	4	58 (54–63)
	M	34	62 (56–69)	<0.01	95	2	60 (54–67)
	F	31	58 (50–65)	<0.01	92	2	57 (49–64)
Any vitamin/mineral	M and F	22	50 (43–57)	<0.01	91	1	49(42–57)
	M	9	50 (41–60)	<0.01	85	2	47 (37–57)
	F	14	52 (43–62)	<0.01	82	1	49 (40–59)
MVM	M and F	44	34 (30–40)	<0.01	96	2	33 (28–38)
	M	21	37 (31–44)	<0.01	90	2	36 (19–32)
	F	25	36 (29–44)	<0.01	88	2	35 (26–44)
Vitamin A	M and F	10	6 (4–7)	<0.01	91	1	6 (4–10)
	M	6	4 (2–8)	<0.01	88	1	5 (2–9)
	F	5	3 (1–10)	<0.01	86	2	5 (2–13)
Vitamin B or B complex	M and F	24	17 (12–23)	<0.01	96	0	17 (12–23)
	M	12	18 (11–28)	<0.01	95	0	18 (11–28)
	F	10	15 (9–23)	<0.01	85	1	17 (11–26)
Vitamin C	M and F	34	32 (26–39)	<0.01	96	1	33 (27–39)
	M	18	34 (25–44)	<0.01	97	0	34 (25–44)
	F	20	31 (25–38)	<0.01	84	8	41 (35–49)
Vitamin D	M and F	10	7 (3–15)	<0.01	96	0	7 (3–15)
	M	5	10 (3–32)	<0.01	94	0	10 (3–32)
	F	3	7 (2–28)	<0.01	86	^a^	^a^
Vitamin E	M and F	24	13 (10–18)	<0.01	94	3	16 (12–21)
	M	10	14 (8–23)	<0.01	92	1	16 (10–25)
	F	7	8 (4–15)	<0.01	79	1	9 (5–17)
Iron	M and F	30	17 (12–23)	<0.01	95	0	17 (12–23)
	M	10	11 (5–24)	<0.01	96	0	11 (5–24)
	F	18	23 (15–34)	<0.01	94	0	23 (15–34)
Calcium	M and F	25	12 (8–18)	<0.01	94	5	15 (11–22)
	M	7	20 (7–47)	<0.01	97	0	20 (7–47)
	F	14	17 (11–25)	<0.01	90	3	21 (14–30)
Zinc	M and F	10	7 (5–10)	<0.01	80	0	7 (5–10)
	M	5	7 (4–12)	0.01	68	0	7 (4–12)
	F	5	5 (2–13)	<0.01	81	2	8 (3–17)
Protein	M and F	33	27 (20–35)	<0.01	98	0	27 (20–35)
	M	20	36 (25–49)	<0.01	78	1	37 (25–50)
	F	15	12 (7–22)	<0.01	95	0	12 (7–22)
Amino acid	M and F	24	15 (12–20)	<0.01	97	0	15 (12–20)
	M	15	15 (9–23)	<0.01	96	0	15 (9–23)
	F	10	10 (3–23)	<0.01	96	0	10 (3–23)
Creatine	M and F	24	14 (10–20)	<0.01	97	0	14 (10–20)
	M	18	17 (11–26)	<0.01	97	3	20 (13–30)
	F	14	3 (1–4)	<0.01	53	5	3 (2–6)
*Echinacea*	M and F	7	12 (4–29)	<0.01	97	0	12 (4–29)
	M	3	11 (3–40)	<0.01	87	^a^	^a^
	F	4	13 (2–60)	<0.01	96	0	13 (2–60)
Ginseng	M and F	9	8 (5–14)	<0.01	96	1	9 (5–15)
	M	5	10 (6–17)	<0.01	89	0	10 (6–17)
	F	5	8 (3–20)	<0.01	94	0	8 (3–20)
Sport drink	M and F	17	28 (18–24)	<0.01	98	0	28 (18–24)
	M	10	44 (24–66)	<0.01	96	0	44 (24–66)
	F	8	35 (22–51)	<0.01	89	0	35 (22–51)
Sport bar	M and F	9	34 (22–47)	<0.01	95	0	34 (22–47)
	M	7	28 (14–56)	<0.01	96	0	28 (14–28)
	F	3	32 (12–62)	<0.01	96	0	32 (12–62)
Ω-3-fatty acid supplement	M and F	6	14 (8–24)	<0.01	94	1	18 (10–28)
	M	4	21 (12–31)	<0.01	91	1	25 (15–39)
	F	3	20 (12–32)	<0.01	79	^a^	^a^
Energy drink^b^	M and F	3	34 (26–43)	<0.01	71	^a^	^a^
Caffeine	M and F	4	29 (16–46)	<0.01	94	1	24 (2–41)
	M	2	20 (16–26)	0.73	0	^a^	^a^
	F	2	21 (16–24)	0.14	54	^a^	^a^

*DS* dietary supplement, *F* females, *M* males, *MVM* multivitamin/multimineral, *SPE* summary prevalence estimate, *S95* *% CI* summary 95 % confidence interval

^a^Not calculated with fewer than four studies

^b^No study reported men and women separately

## Discussion

This review illustrates the difficulty in obtaining a comprehensive description of the prevalence of dietary supplement use in athletes. Studies on this topic have used different data collection methods (e.g., dietary surveys, interviews, questionnaires), collected data on different dietary supplements, and used different reporting timeframes. The methodological quality of the studies was generally very low, with only 34 % of studies acquiring even half the available points on the Loney et al. [28] checklist. Methodological quality of the studies published after the 1990s improved slightly, but average improvement was only an additional 6 % of available points (37 vs. 43 %). Using meta-analysis, an attempt was made to summarize the data by sex and sport, but few studies reported use of the same dietary supplements, and where this was the case the prevalence values in many individual studies varied widely (low homogeneity), resulting in broad S95 % CIs. Nonetheless, the tables in the present study provide a comprehensive summary of available data on the use of dietary supplements by athletes.

When comparisons were made between elite and non-elite athletes, the SPEs suggested that elite athletes tended to use dietary supplements to a greater extent than the non-elite athletes. However, prevalence ranges among studies were still wide, demonstrating low homogeneity. Further, this comparison was complicated by differences in the types of sports that might be included in the two groups. Athletes involved in different sports might be expected to use different dietary supplements, although the combined data on specific dietary supplements in specific sports was too sparse to test this hypothesis (Table 2). The sex comparisons within the elite/non-elite groups might be more appropriate because the comparisons involve similar (although not identical) sports. However, the prevalence values still varied widely and the SPEs lacked homogeneity. Nonetheless, examining the individual study prevalences and the SPEs within the elite or non-elite groups suggested little difference between men and women in overall prevalence of dietary supplement use.

Like comparisons by sport and elite/non-elite status, the attempt to summarize the sex-specific prevalences of particular dietary supplements for all studies on athletes suffered from a lack of homogeneity. Nonetheless, SPEs appeared to be similar between men and women, with a few exceptions. Compared with men, a larger proportion of women appeared to use iron supplements. A much larger proportion of active women appear to be iron deficient compared with active men [178], and the recommended daily allowances for iron are more than twice as high for premenopausal women (18 mg/day) than for men (8 mg/day) [179]. This may lead more athletic women to supplement with iron to enhance health and/or performance. Consumption of iron supplements was associated with a lower prevalence of iron deficiency among adult women in a US national survey [180]. Protein and creatine were also taken by a larger proportion of men than women, possibly because of differences in rationales for using dietary supplements. When men and women were queried separately on their reasons for using dietary supplements within the same study, men reported the development of strength and/or muscle mass as a higher priority than did women [7, 11, 19]. Protein and creatine supplementation have both been demonstrated to increase strength and lean body mass [181, 182].

Table 5 shows summary data from the National Health Interview Surveys (NHIS) [183–185] and the National Health and Nutrition Surveys (NHANES) [186–189], both of which are nationally representative samples of the general US population. Both surveys observed a secular (temporal) trend, indicating that dietary supplement use has been increasing over time, although the most recent NHANES data suggested a leveling off in use [188, 189]. The increase in dietary supplement use is in consonance with data showing an eightfold increase in commercial dietary supplement sales from 1994 to 2012 [3, 4]. In the present review, studies were compiled by publication date in an attempt to determine whether a temporal trend was present, but no such trend could be discerned. Even the earliest studies in the literature demonstrated that a very large proportion of athletes were using dietary supplements [54, 56, 83, 95, 139, 147].

**Table 5:** Summary data on dietary supplement use in US national surveys

Survey	Study	Survey year(s)	*n*	Reporting timeframe	Prevalence (%)					
					Any VM		MVM		Vitamin C	
					M	W	M	W	M	W
National Health Interview Survey (NHIS)	Subar and Block 1990 [183]	1987	9160 M, 12,920 F	Daily use	19	27	15	20	7	8
	Slesinski et al. 1995 [184]	1992	5120 M, 6885 F	Daily use	20	27	17	22	7	8
	Millens et al. 2004 [185]	2000	34,085 M and F	Daily use	29	39	24	33	10	12
National Health and Nutrition Survey (NHANES)	Koplan et al. 1986 [186]	1976–1980	5915 M, 6588 F	Use ≥1 time/wk	30	40	ND	ND	ND	ND
	Balluz et al. 2000 [187]	1988–1994	33,905 M and F	Use in last month	35	44	ND	ND	ND	ND
	Radimer et al. 2004 [188]	1999–2000	2260 M, 2602 F	Use in last month	46	57	32	38	12	13
	Kennedy et al. 2013 [189]	2007–2008	3364 M and F	Use in last month	42	54	ND	ND	ND	ND

*VM* vitamin and/or mineral, *MVM* multivitamin/multimineral, *M* males, *F* females, *ND* no data, *wk* week

Additionally, Table 5 includes summary data on multivitamins/multiminerals and vitamin C, mostly from the NHIS [183–185], but including one NHANES survey [188]. There are some limitations in comparing these national survey data with those of the athletes. The NHIS survey asked individuals about daily use of supplements, and the NHANES asked about use in the last month. In the meta-analyses of athletes presented here (Tables 2, 3, 4) the reporting timeframe included ‘current’, ‘usual’, or ‘regular’ use. The athlete data were also collected over a much greater range of years than the NHIS/NHANES data, and the temporal trends differed, as noted above. Despite these limitations, a comparison of the data suggested that prevalence of use of any vitamins/minerals and multivitamins/multiminerals was somewhat higher, and that of vitamin C much higher among athletes than the general US population. Similar to the athlete data, a recent systematic review showed that the prevalence of multivitamin/multimineral and vitamin C use was higher in the military than in civilian samples [190]. Military and athletic groups may be more likely to use dietary supplements because of a greater concern with health and performance [10, 35, 53, 149, 191–196]. For example, consider protein supplements. Protein supplementation in combination with resistance training augments gains in fat-free mass [182]. National surveys in the USA indicate that only 1 % of the general population uses amino acid supplements [197]. In contrast, the athlete data suggests a much larger prevalence of use (Table 4), as does the more limited military data [190].

Use of herbal supplements could be high in some athletic subgroups, especially among elite athletes. For example, use of ginseng ranged from 16 to 51 % in a study of Korean Olympic athletes [144, 153] and 27 % in a study of Saudi Arabian professional soccer players [68]. *Echinacea* was used by 60 % of professional surfers [123], 39 % of elite Australian swimmers [58], and 28 and 44 % of elite male and female, respectively, figure skaters [15]. Nonetheless, in most groups of athletes, 10 % or fewer used herbal supplements [10, 19, 23, 49, 67, 80, 101, 121, 128, 159, 198]. In US national surveys, the most commonly used herbal supplements were *Echinacea*, garlic, *Ginkgo biloba*, ginseng, and Saint John’s Wort, with most use prevalences well below 5 % [185, 197, 199]. Since the studies reviewed here were conducted over a long period of time, the popularity of different supplements may have changed over time as they have in the general US population [199, 200].

We suspected there would be differences among studies based on nationality because of cultural and/or socioeconomic conditions. Some differences were noted in the use of ginseng, since Korean and Saudi Arabian athletes had a higher prevalence of use of this herbal supplement [68, 144, 153] than other nationalities [9–12, 15, 19, 49, 58, 80, 131, 132, 141, 149, 152, 159, 198]. However, no other major differences were noted; there was a wide range of dietary supplement usages regardless of nationality.

## Suggestions for Improving Research on Dietary Supplement Use in Athletes

Because of the lack of homogeneity in dietary supplement use prevalences among athletes, the most appropriate way to conclude this review is to offer suggestions for improving the collection of dietary supplement data. Future studies on athletes and other groups should consider five major issues. First, the definition of dietary supplement should be clearly stated. The legal definition provided by the DSHEA of 1994 [2] can serve as a standard. Second, studies should be specific about the types of dietary supplements used by study participants. Reporting in general categories like ‘antioxidant’, ‘energy’, ‘herbal’, ‘bodybuilding’, and the like may be useful for some purposes but does not provide the specificity needed for comparisons across studies or the identification of the specific dietary supplements that are being used by athletes. Third, the reporting timeframe should be specific and include several periods. The most useful reporting timeframes appear to be daily, 2–6 times/week, 1 time/week, and <1 time/week. Fourth, the response rate (those volunteering/those solicited) should be specified and, if possible, characteristics of respondents and non-respondents described so that possible bias can be assessed. Finally, studies are needed that use the same experimental methods to compare dietary supplement use across a wide variety of sports so sport-specific differences and temporal trends can be established. Following these guidelines will assist in providing more accurate and comparable data on dietary supplement use in specific athletic populations.

## Conclusions

When dietary supplement use was compiled by sport, elite versus non-elite athletic status, and supplement type, there was high variability in use prevalence among studies. Elite athletes appeared to use dietary supplements much more than their non-elite counterparts. For most dietary supplements, use prevalence appeared similar for men and women, with the exception of iron, creatine, protein, and vitamin E. Suggested improvements in future studies include (1) defining dietary supplements in surveys and dietary evaluations; (2) querying for very specific dietary supplements; (3) using a variety of reporting timeframes (e.g., daily, 2–6 times/week, 1 time/week and <1 time/week); (4) reporting the sampling frame, number of individuals solicited, and number responding; (5) reporting characteristics of volunteers (and non-volunteers, if available); and (6) using similar methods on several occasions to examine possible temporal trends among athletes.

## Electronic supplementary material

Supplementary material 1 (DOCX 285 kb)
