# Supplementary material for: Prevalence of Dietary Supplement Use by Athletes: Systematic Review and Meta-Analysis
Source: Sports Med. 2015 Oct 6;46:103–23. doi: 10.1007/s40279-015-0387-7 (PMC4697915; doi:10.1007/s40279-015-0387-7)
Supplement: Supplementary file 1 — Supplementary material 1 (DOCX 285 kb) [file 40279_2015_387_MOESM1_ESM.docx]

Electronic Supplementary Material Table S1. Methods in investigations of dietary supplement use by athletes

| Study | Participants | Methods for collecting supplement information | Reporting timeframe^a^ | Proportion (%) of initial sample participating in study^b^ | Methodological quality score (%)^C^ |
| --- | --- | --- | --- | --- | --- |
| Bobb et al. 1969[116] | 12 ♂ basketball players, 10 ♂ ice hockey players, 6 ♂ swimmers, collegiate athletes | Interview by students in University Food & Nutrition Department | Use in last 2 months | Not specified | 16 |
| Steel 1970[139] | 66 ♂ & 14 ♀ Australian Olympic athletes, ~17 sports | 7-day dietary record & questionnaire | Current use | 55 | 21 |
| Bright-See et al. 1978 [83] | 51 ♀ ballet students, National Canadian Ballet, aged 10-18 yr | Questionnaire with items on DSs | Daily Use | Not specified | 29 |
| Werblow et al. 1978[146] | 94 ♀ collegiate athletes, 10 sports | Questionnaire with items on DSs | Current use in “general diet” | 75 | 42 |
| Houston 1980[54] | 8 ♂ & 12 ♀ elite Canadian swimmers at a training camp | 3-day weighted food intake record | Current use, 3-day period | 49 | 46 |
| Barry et al. 1981[140] | 115 ♂ & 28 ♀ nominated for 1980 Moscow Olympics from Ireland | 3-day weighted food intake record | Current use, 3-day period | ~90 | 79 |
| Adams et al. 1982[55] | 19 ♀ collegiate swimmers | 1-day dietary recall obtained once a week over 7 weeks | Current use, 7 day period | Not specified | 29 |
| Short & Short 1983[42] | 40 ♂ football players, 38 ♂ wrestlers, 12 ♂ basketball players, collegiate athletes | 1-day dietary recall (football & basketball); 3-day to 2-week dietary intake record (wrestlers) | Current use, 1 day period (football & basketball) or longer (wrestlers) periods | ~56 (football); not specified for other athletes | 25 |
| Grandjean 1983[127] | 69 world-class amateur & professional athletes | Not stated | Not clear | Not specified | 8 |
| Calabrese et al. 1983[84] | 25 ♀ professional ballet dancers, Cleveland Ballet Company | 3-day food intake record | Current use in 3-day period | Not specified | 33 |
| Moffatt1984[90] | 13♀ HS gymnasts (aged 15±4 yr), Washington State Champions in 1982 | 2, 3-day food intake records including one weekend day | Current use, 6-day period | Not specified | 21 |
| Douglas 1984[157] | 515 ♂ & 425 ♀ athletes from 10 Connecticut HSs, 18 sports | Questionnaire including item related to vitamin use | Regular use | Not specified | 46 |
| Parr et al. 1984[170] | 1432 ♂ & 1,547 ♀ athletes from across the US | Questionnaire with items on DSs | Not clear | Not specified | 38 |
| Campbell & MacFadyen 1984[56] | 38 ♂ & 63 ♀ adolescent competitive swimmers (aged >13 yr) in swim clubs in Winnipeg, Canada | Questionnaire with items on DSs use | Current use, training period | 84 | 50 |
| Braisted et al. 1985[86] | 44 ♀ adolescent ballet dancers (aged 16±2 yrs) | Questionnaire including items on DSs | Not clear | Not specified | 25 |
| Grandjean 1985[128] | ~150 elite athletes, 12 sports | Not clear (reported as “comprehensive dietary data”) | Not clear | Not specified | 17 |
| Benson et al. 1985[87] | 92 ♀ adolescent ballet dancers enrolled In 6 professional schools (ages 12-17 yr) | 3-day diet history with 1 weekend day | Current use 3-day period | Not specified | 29 |
| Cohen et al. 1985[85] | 10 ♂ & 12 ♀ professional ballet dancers, members of the American Ballet Theater | Questionnaire with items on DSs | Daily use | Not specified | 33 |
| Deuster et al. 1986 & Singh et al. 1986 & 1990[36-38] | 45-51 ♀ runners competing in first women’s Olympic Marathon Trial in 1984 | 3-day dietary record with 1 weekend day | Current use, 3-day period | 22-25 | 58 |
| Lampe et al. 1986[94] | 9 ♀ marathon runners in training | 3- day food diary | Current use, 3-day period | Not specified | 33 |
| Barr 1986[95] | 104 ♀ marathon runners & 105 ♀ members of a fitness club | Questionnaire with items on DSs | Daily use | 58 | 46 |
| Loosli et al. 1986[91] | 97 ♀ adolescent gymnasts (aged11-17 yr) in 6 gymnastic schools in San Francisco Bay area | 3-day dietary record with 1 weekend day | Current use, 3-day period | Not specified | 46 |
| Slavin et al. 1986[119] | 36 ♀ elite cyclists & 76♀ recreational cyclists | Questionnaire with items on DSs | Not clear | Not Specified | 21 |
| Thibault et al. 1986[96] | 1123 runners registered for a Montreal marathon | Questionnaire with items on DSs | Current use, before or after marathon | 48 | 54 |
| Khoo et al. 1987[98] | 19 ♂ & 10 ♀ triathletes competing in 1983 Hawaiian Ironman | 3-day diet record in week before the race | Current use, 3 day period | 30 | 33 |
| Barr 1987[147] | 70 ♀ collegiate athletes , 8 sports | Questionnaire with items on DSs | Not clear | 66 | 63 |
| Evers 1987[103] | 21 ♀ enrolled in intermediate & advanced university dance classes | 3-day food record with 1 weekend day | Current use, 3-day period | Not specified | 29 |
| Faber 1987[69] | 74 ♂ body-builders recruited from 14 gyms in South Africa | 7-day diet record with separate form to record DSs | Current use, 7 day period | Not specified | 33 |
| Burke & Read 1988[62] | 54 ♂ elite Australian soccer players | 7-day food diary | Current use, 7-day period | Not specified | 42 |
| Schulz 1988[79] | 66 ♂ & 61 ♀ basketball players & cross-country runners; 24 ♂ & 66 ♀ exercisers | Questionnaire focused on DS use | Current use | Not specified | 29 |
| Clark et al. 1988[110] | 93 ♀ elite middle- & long-distance runners who competed nationally | Questionnaire with items on DSs | Regular Use | 81 | 46 |
| Nowak et al. 1988[78] | 16 ♂ & 10 ♀ collegiate basketball players, NCAA Division II | 3-day food diary, weekdays only | Current use, 3 day period | Not specified | 42 |
| Lamar-Hildebrand et al. 1989[72] | 10 ♀ competitive bodybuilders | 4, 3-day food records, 1 weekend day each | Current use, 12-day period | Not specified | 29 |
| Krowchuk et al. 1989[158] | 212 ♂ & 83 ♀ HS athletes in suburban Cleveland area | Questionnaire with items on DSs | Any use now or in past | 99 | 50 |
| Snyder et al. 1989[122] | 10 ♂ & 7 ♀ elite speed skaters | 3-day food intake record with 1 weekend day | Current use, 3-day period | Not specified | 29 |
| Sandoval et al. 1989[70] | 5 ♂ & 6 ♀ body builders | 3-day dietary record | Current use, 3-day period | Not specified | 33 |
| Nieman et al. 1989[97] | 291 ♂ & 56 ♀ marathon runners competing at the Los Angeles Marathon | 3-day food record | Current use, 3-day period | 7 | 71 |
| Heyward et al. 1989[71] | 7 ♂ & 12 ♀ elite bodybuilders | 3-day dietary record | Current use, 3-day period | Not specified | 33 |
| Worme et al. 1990[99] | 50 ♂ & 21 ♀ competitive triathletes | 3-day dietary record with 1 weekend day | Daily use, 3 day period in usual training | 16 | 50 |
| Frusztajer et al. 1990[88] | 20 ♀ classical ballet dancers | 2-day dietary recall & quantitative food frequency questionnaire | Current use | Not specified | 33 |
| Kleiner et al. 1990[73] | 19 ♂ & 8 ♀ elite bodybuilders competing in 1988 USA Body Building Championship | 7-day food record in week before competition | Current use, 7-day period | 26 | 41 |
| Pate et al. 1990[111] | 103 ♀ runners recruited from runner’s clubs or running events | 2, 3-day dietary intake records including 1 weekend day each | Current use, 6 day period | Not specified | 33 |
| Ersoy 1991[92] | 20 ♀ Turkish child gymnasts, aged 12±1 yrs | 3-day dietary record with 1 weekend day | Current use, before competition | Not specified | 29 |
| Burke et al. 1991[63] | 25 ♂elite triathletes, 19 ♂elite marathon runners, 56 ♂ elite soccer players & 19 ♂ elite weightlifters, Australia | Interview followed by 7-day food intake record | Regular or daily use | Not specified | 46 |
| Faber & Benade 1991[125] | 20 ♂ & 10 ♀ elite field athletes (discus, hammer, javelin, shotput) participating in 1988 national championship | Questionnaire including items on DSs | Not clear | Not specified | 25 |
| Hawley & Williams 1991[57] | 9 ♂ & 11 ♀ young competitive swimmers, aged ~13±2 yr | 4-day dietary recall | Current use, 4-day period | Not specified | 29 |
| Soper et al. 1992[118] | 535 aerobic dance instructors | Questionnaire including items on DSs | Regular use | 54 | 42 |
| Frederick & Hawkins 1992[104] | 14 ♀ collegiate dancers & 13 ♀ collegiate track team members , Indiana State University | Questionnaire including items on DSs | Daily use | Not specified | 33 |
| Stensland & Sobal 1992[201] | 17 ♂ & 89 ♀ dancers recruited in Baltimore/Washington DC area , ballet, jazz, & modern dance, professional & amateur | Questionnaire with item on vitamin/mineral DS use | Any use | Not specified | 38 |
| Fogelholm et al. 1992[129] | 427 ♂ Finnish athletes, 11 sports | Food frequency questionnaire | Use in month prior to study | Not specified | 50 |
| Jacobson & Aldana 1992[43] | 533 ♂ & 259 ♀ athletes from 11 NCAA Division IA universities, 8 sports | Questionnaire with items on DS use | Regular use | Not specified | 46 |
| Singh et al. 1993[106] | 15 ♂ & 2 ♀ ultra-marathoners competing in 1990 Old Dominion 100-mile race | 4-day dietary recall with 2 weekend days | Usual & prerace use in 4- day period | 23 | 54 |
| Bazzarre et al. 1993[171] | 61 ♀ & 30 ♀ recreational athletes recruited from sporting goods stores, & athletic facilities | 7-day food record | Current use, 7-day period | Not specified | 33 |
| Oppliger et al. 1993[82] | 713 ♂ wrestlers recruited from 45 Wisconsin HSs | Questionnaire with items on DSs | Current use | 82 (% of HSs responding) | 83 |
| Walberg-Rankin et al. 1993[75] | 6 ♀ bodybuilders from clubs in Virginia & Maryland | 1-day dietary record recorded 8 times over 49-day period before & after competition | Current use, 8-day period | Not specified | 33 |
| Linseisen et al. 1993[74] | 13 ♂ amateur German bodybuilders | 14-day weighted dietary record | Current use, 14-day non-competitive period | Not specified | 33 |
| Sobal 1994[44] | 430 ♂ & 312 ♀ athletes from 9 rural HSs, >16 sports | Questionnaire on use of vitamin or mineral DSs | Current use | Not specified | 46 |
| Musaiger 1994[130] | 304 elite Bahraini athletes from 14 athletic clubs in Bahrain,4 sports | Interview with items on DS | Current use | Not specified | 54 |
| Brill & Keane 1994[6] | 210 ♂ & 99 ♀ bodybuilders competing in Florida in the 1991/1992 season | Questionnaire focused on DSs | Bulking phase of body building (training to increase muscle mass) | 25 | 71 |
| Massad, 1995[159] | 302 ♂ & 205 ♀ athletes & non-athletes (24% of sample) in 6 Indiana HSs,~34 sports | Questionnaire focused on DSs | Current use, weekly to daily | Not specified | 50 |
| Andersen et al. 1995[76] | 49 ♂ body builders competing in the All Natural Middle Atlantic States Body Building Championship | Questionnaire with items on DSs | Current use, in training | 92 | 63 |
| Peters & Goetzsche 1997[107] | 150 ♂ & 23 ♀ ultramarathoners competing in 90-km 1993 Comrades Marathon in South Africa | 1-day dietary recall | Current use, in training | Not specified | 46 |
| Beals & Manore 1998[172] | 48 ♀ athletes, 24 with eating disorders, 9 sports | 7-day weighted food intake record | Current use, 7-day period | NA (case-control study) | 38 |
| Ziegler et al. 1998[113] | 12 ♂ & 16 ♀ adolescent figure skaters training at the US Olympic Training Center, aged ~15±2yr | 4-day diet record including 2 weekend days | Current use, 4-day period | Not specified | 46 |
| Jonnalagadda et al. 1998[93] | 33 ♀ elite artistic gymnasts, US National Women’s Artistic Gymnastic Team | 3-day food intake record with 1 weekend day | Current use, 3-day period | Not specified | 42 |
| Felder et al. 1998[123] | 10 ♀ elite professional surfers | Questionnaire including items on DSs | Current use, in training | Not specified | 33 |
| Rosen 1999[115] | 33 ♀ power athletes (weightlifting, boxing, track & field), 19 ♂ & 14 ♀ alpine skiers, 17 ♂ & 17 ♀ X-C skiers, Norwegian National Teams | Questionnaire with items on DSs | Regular use | Not specified | 33 |
| LaBotz & Smith 1999[45] | 739 ♂ & ♀collegiate athletes at University of North Carolina 1997/1998 academic year | Questionnaire on creatine use | Use at any time | 92 | 58 |
| Krumbach et al. 1999[7] | 266 ♂ & 145 ♀ collegiate varsity athletes at University of Nebraska (Lincoln), 1997 | Questionnaire on vitamin/mineral DS use | Usual use | ~90 | 54 |
| Kim & Keen 1999[160] | 926 ♂ & 429 ♀ HS athletes enrolled in 13 athletic HSs in South Korea, >15 sports | Questionnaire on vitamin/mineral DS use | Regular use >1 month in previous year | 61 | 75 |
| Swirzinski  2000[8] | 170 ♂ HS football players from 5 Midwest HSs | Questionnaire focused on ergogenic DS | Current use | 99 | 54 |
| Greenwood et al. 2000[46] | 156 ♂ & 63 ♀ at NCAA Division I college, 5 sports each sex | Questionnaire focused on creatine & other DS use | Current use | 95 | 58 |
| Smith & Dahm 2000[47] | 182 ♂ & 146 ♀ HS athletes in Minnesota | Questionnaire focused on creatine & other DS use | Use at any time | Not specified | 33 |
| Sheppard et al. 2000[163] | 85 ♂ & 11 ♀ members of health clubs | Questionnaire focused on creatine & other DS use | Current Use | 33 | 33 |
| Kanayama et al. 2001[164] | 334 ♂ & 177 ♀ training in 5 Boston gyms | Questionnaire focused on ergogenic DSs | Use >6 months in last 3 years | ~50 | 29 |
| Jacobson et al. 2001[148] | 205 ♂ & 125 ♀ collegiate athletes from 16 NCAA Division 1A universities, 11 sports | Questionnaire with items on DSs | Use during college athletic career | 41 | 38 |
| Jonnalagadda 2001[50] | 31 ♂ freshman football players, NCAA Division 1 School | Questionnaire with items on DSs | Current use | Not specified | 29 |
| Berglund 2001[141] | Swedish Athletes in Olympic Games: Atlanta 1996 (n=183); Nagano 1998 (n=105); Sydney 2000 (n=157) | Questionnaire focused on medications & DS | Current use, before Olympic games | 100 | 58 |
| Ray et al. 2001[161] | 469 ♀ & 205 ♀ adolescent athletes (ages 13-19 yrs) from 11 private & public schools in Tennessee & Georgia | Questionnaire focused on creatine use | Current use | Not clear | 33 |
| Metzl et al. 2001[48] | 604 ♂ & 492 ♀ middle school & HS athletes in 5 suburban New York City schools, 15 sports | Questionnaire focused on creatine use | Use at any time | Not specified | 33 |
| Baylis et al. 2001[58] | 40 ♂ & 37 ♀ Australian elite swimmers, national & potential national level | Questionnaire focused on DSs | Use in training or competition in last 6 months | 48 | 33 |
| Mason et al. 2001[49] | 495 ♂ football players & 407 ♀ volleyball players from 20 Iowa HSs | Questionnaire focused on DSs | Use at any time | Not specified | 29 |
| Mullins et al. 2001[126] | 17 ♀ elite heptathletes (100-m hurdles, high jump, shot put, 200-m dash, long jump, javelin, 8000-m run) | 4-day dietary record with 1 weekend day | Current use, 4-day period | Not specified | 38 |
| Ziegler et al. 2002[114] | 18 ♀ young competitive figure skaters, aged 14-16 yr | 3-day food record including 1 weekend day | Use at any time | Not specified | 42 |
| Schroder et al. 2002[80] | 55 ♂ elite Spanish basketball players from 7 separate teams | Questionnaire focused on DSs | Use at any time | 71 | 46 |
| Beals 2002[81] | 23 ♀ elite adolescent volleyball players (aged 16±1 yr), nationally ranked | 3-day weighted food record with 1 weekend day | Current use, 3-day period | Not specified | 42 |
| Sundgot-Borgen et al. 2003[9] | 666 ♂ & 556 ♂ elite Norwegian athletes on national senior & junior teams | Questionnaire focused on DS use | Current use | 83 | 71 |
| Ziegler et al. 2003[15] | 46 ♂ & 59 ♀ elite figure skaters, competing in 2001 US Figure Skating National Championships | Questionnaire focused on DSs | Current use | Not specified | 38 |
| Ama et al. 2003[64] | 1037♂ & 79 ♀ amateur soccer players in Yaounde Cameroon | Questionnaire that included item on Vit C | Not Clear | 74 | 38 |
| Slater et al. 2003[131] | 85 ♂ & 75 ♀ Singaporean athletes competing on national level, 30 sports | Questionnaire focused on DSs | Use in training in last 12 months | 29 | 50 |
| Corrigan & Kazlauskas 2003[142] | 2,758 athletes selected for doping control at the Sydney Olympic games (2000), 32 sports | Questionnaire on medications & DSs (Doping Control forms for Olympic Games) | Current use, 3-day period | 27 | 42 |
| Herbold 2004[149] | 162 ♀ collegiate athletes at 2 universities, 9 sports | Questionnaire focused on DSs | Any use during training or sports season | 71 | 54 |
| Beitz et al. 2004[173] | 1015 ♂ & 1271 ♀ Germans engaged in sport at least once a week | Interview including items on vitamin & mineral DSs | Current use ≥ 1 time/wk | Not specified | 67 |
| Nogueira & DaCosta 2004[100] | 29 ♂ & 9 ♀ competitive Brazilian triathletes | Food frequency questionnaire | Use in last 3 months | Not specified | 42 |
| Morrison 2004[10] | 182 ♂ & 40 ♀ using gyms on Long Island NY ≥4 times/wk for ≥ 1 year | Questionnaire focused on DSs | Regular use | Not specified | 29 |
| Froiland 2004[11] | 115 ♂ & 88 ♀, at an NCAA Division I University, 15 sports | Questionnaire focused on DSs | Current use | 55 | 29 |
| Burns 2004[150] | 118 ♂ & 118 ♀ athletes at 8 NCAA Division 1 Universities, 16 sports | Questionnaire focused on DSs | Current use | 66 | 50 |
| Paschoal & Amancio 2004[59] | 8 ♂ elite Brazilian swimmers competing at national level | Not clear, likely questionnaire | Use in last 3 months | Not specified | 38 |
| Bartee et al. 2004[162] | 1014 ♂ & 721 ♀ HS athletes in large metropolitan area in Southwest US | Questionnaire focused on ergogenic DSs | Not clear | Not specified | 58 |
| Nieper 2005[16] | 20 ♂ & 12 ♀ UK Junior National Track & Field Athletes (aged 18 yr) | Questionnaire focused on DSs | Occasional to daily use | 94 | 42 |
| Waddington et al. 2005[65] | 706 ♂ professional English soccer players | Questionnaire with items on DSs | Not clear | 25 | 50 |
| Kristiansen 2005[151] | 120 ♂& 89 ♂Canadian collegiate athletes, ~10 sports | Questionnaire focused on DSs | Use in last month | 52 | 63 |
| Cole et al. 2005[51] | 28 ♂ collegiate football players, NCAA Division I | 2, 3-day diet records | Daily use | Not specified | 29 |
| Erdman 2006; 2007[34, 35] | 314 ♂ & 268 ♀ elite from national Canadian teams or developmental elite teams, 27 sports | Questionnaire focused on DSs | Use in last 6 months | 69 | 63 |
| Scofield & Unruh 2006[52] | 99 ♂ & 34 ♀ HS athletes in central Nebraska, 12 sports | Questionnaire focused on DSs | Current Use | 23 | 16 |
| Huang et al. 2006[198] | 121 ♂ & 136♀ Canadian athletes competing in Atlanta Olympic Games (1996); 150 ♂& 150 ♀ Canadian athletes competing in Sydney Olympic Games (2000) | Interview with items on DSs | Current use | 97 | 58 |
| Striegel et al. 2006[174] | 414 ♂ & 184 ♀ master athletes (aged 50±10 yrs) competing in World Master Athletic Championships Indoors, 2004 | Questionnaire focused on DSs | Current use | 38 | 46 |
| Bent & Marsh 2006[117] | 139 college hockey players | Questionnaire focused on use of ergogenic substances | Past 30 days | 100 | 42 |
| Petroczi et al.2007[12, 39-41] | 866-874 UK athletes responding to the “UK Sports 2005 Drug Free Survey”, ~37 sports | Questionnaire focused on DSs | Any use current or in past | 29 | 42 |
| Chlopicka et al. 2007[165] | 113 ♂ & 37 ♀ who exercise in gyms in Krakow, Poland area | Questionnaire focused on DSs | Any Use | Not specified | 13 |
| Duellman 2008[53] | 61 ♂ HS football players in a small Midwest US town | Questionnaire focused on protein supplementation use | Current use | Not specified | 33 |
| Petroczi et al. 2008[132] | 260 ♂& 133 ♀ elite young British athletes, 34 sports, aged 12-21 yr. | Questionnaire focused on DSs | Any use current or in past | 24 | 54 |
| Tscholl et al. 2008[66] | 736 ♂ professional soccer players competing in each of 2 years in the World Cup 2002 & 2006 | Interview including items on DSs | Current use, 72 hr prior to match | 100 | 63 |
| Knechtle et al. 2008[108] | 20 ♂ ultra-distance runners planning to compete in 1,200 km race | Questionnaire focused on vitamin & mineral use | Current use, month before race | 63 | 13 |
| Sekulic et al. 2008[105] | 22 ♂ & 21 ♀ Serbian sports dancers | Questionnaire with items on DSs | Current use | Not specified | 42 |
| Soric et al. 2008[202] | 23 ♀ Croatian gymnasts & 16 ♀ Croatian ballet dancers | Food frequency questionnaire | Use on regular basis | Not specified | 42 |
| Tsitsimpikou et al. 2009[143] | 2463 Doping Control Official Records & 1779 Therapeutic Use Exception records from the 2004 Athens Olympic Games | Questionnaire with items on DSs | Current use, around Olympic Games | Not specified | 42 |
| Tian et al. 2009[152] | 35 ♂ & 47 ♀ Singaporean collegiate athletes, 16 sports | Questionnaire focused on DSs | Use in last 12 months | 43 | 38 |
| Lun et al. 2009[133] | 201 ♂ & 114 ♀ elite Canadian athletes, national/international level, 36 sports | 3-day dietary record | Current use, 3-day period | Not specified | 54 |
| Braun et al. 2009[134] | 77 ♂ & 87 ♀ elite German athletes, 26 sports | Questionnaire focused on DSs | Any use current or in the past | 72 | 46 |
| Tscholl et al. 2010[135] | 1,947 ♂ & 1,940 ♀ elite track & field athletes worldwide who filled out doping control forms | Questionnaire including items of DSs | Current use, last 7 days | Not specified | 38 |
| Knez & Peake 2010[101] | 24 ♂ & 13 ♀ triathletes recruited from internet Ironman triathlon discussion site | 7-day food diary | Current use, 7 day period | Not specified | 42 |
| Goston 2010[17] | 526 ♂ & 576 ♀ gym participants in 50 Brazilian gyms | Questionnaire focused on DSs | Not clear | Not specified | 58 |
| DeSilva et al. 2010[136] | 73 ♂ & 40 ♀ elite Sri Lankan athletes selected for 2006 South Asian Federation Games, ~5 sports | Interview focused on DSs | Use in last 12 months | 88 | 46 |
| Dascombe 2010[18] | 36 ♂ & 36 ♀ elite Australian athletes in state-based sport institute, 8 sports | Interview & questionnaire on DSs | Current use | Not specified | 13 |
| Zenic et al. 2010[89] | 21 ♀ Croatian ballet dancers from national theater, 25 ♀ Croatian sport dancers, 23 ♀ Croatian synchronized swimmers | Questionnaire with items on DSs | Not clear | Not specified | 38 |
| Kondric et al. 2010[124] | 50 ♂ & 29 ♀ elite Slovenian table tennis players in national competition 2008-2009 | Questionnaire with items on DSs | Current Use | 100 | 67 |
| Kim et al. 2010[13] | 128 ♂ & 100 ♀ elite Korean athletes, 14 sports | Questionnaire focused on DSs | Current use | Not specified | 25 |
| Nazni et al. 2010[137] | 102 ♂ Indian collegiate athletes, 3 sports | Questionnaire with items on DSs | Current use | Not specified | 42 |
| Sanchez-Oliver et al. 2011[166] | 260 ♂ & 155 ♀ using gyms in Seville, Spain | Questionnaire focused on protein use | Current use | Not specified | 54 |
| Karimian et al. 2011[77] | 250 ♂ & 250 ♀ bodybuilders from 30 bodybuilding clubs in Iran | Questionnaire focused on DSs | Current use | Not specified | 50 |
| Tsitsimpikou et al. 2011[167] | 193 ♂ & 136 ♀ recreational athletes from 11 gyms in Athens, Greece | Questionnaire focused on DSs | Current use | 30 | 58 |
| Bianco 2011[168] | 127♂ & 80 ♀ performing strength training in gyms in Palermo, Italy | Interview focused on protein supplementation | Current use | 26 | 58 |
| Heikkinen et al. 2011[32, 33] | 261 ♂ & 185 ♀ Finnish Olympic Athletes in 2002; 218 ♂ &154 ♀ Finnish Olympic Athletes 2009 | Questionnaire focused on DSs | Use in previous 12 months | 90 | 71 |
| Beis et al. 2011[112] | 8 ♂ & 2 ♀ elite Ethiopian distance runners in training for major competitions | 7-day weighted food intake record | Current use, 7-day period | Not specified | 29 |
| Kim 2011[144] | 100-128 ♂ & 82-100 ♀ Korean Olympic Team competing in Beijing Olympics (2008), 11 sports each sex | Questionnaire focused on DSs | Use in 6 months before Olympics | 98 | 63 |
| Kim et al. 2011[153] | 343 ♂ & 136 ♀ elite Korean collegiate athletes, 21 sports | Questionnaire focused on DSs | Current use, in training period | 98 | 67 |
| Russell & Pennock 2011[67] | 10 ♂ UK professional soccer players , youth department team (aged 17±1 yr) | 7-day food intake record | Current use, 7-day period, during competitive season | Not specified | 21 |
| Walsh et al. 2011[120] | 203 ♂ Irish senior schoolboy rugby players | Questionnaire with items on DSs | Current use | Not specified | 50 |
| Dolan et al. 2011[102] | 207 ♂ & 194 ♀ triathletes | Questionnaire with items on DSs | Current use | Not specified | 54 |
| Lazic et al. 2011[23] | 657 ♂ & 255 ♀ elite Serbian athletes, 34 sports | Doping control forms collected over 3 years (2006-2008) | Last 3 days | Not specified | 46 |
| Slattery et al. 2012[60] | 4 ♂ & 2 ♀ elite Australian swimmers participating in intensified training program | 4-day food recall | Any use | Not specified | 17 |
| Lun 2012[19] | 163 ♂ & 277 ♀ elite Canadian athletes recruited at Canadian Sports Centers after workshops, >10 sports | Questionnaire focused on DSs | Use in last 6 months | Not specified | 33 |
| Diehl 2012[145] | 638 ♀ & 500 ♂ German athletes competing in Winter or Summer Olympics1992 or 1995, 51 sports | Questionnaire focused on DSs | Daily use | 58 | 54 |
| Rodek et al. 2012[121] | 39 ♂ & 5 ♀ members of Croatian National Sailing Team | Questionnaire with items on DSs | Use occasionally or often | Not specified | 29 |
| Sato et al. 2012[138] | 26 ♂ & 49 ♀ elite young (aged 13-18 yr) Japanese athletes participating in the youth Olympics in Singapore, 12 sports | Questionnaire followed by interview on DS use | Use in last year | 100 | 33 |
| Hoffman & Fogard 2012 [109] | 392 ♂ & 97 ♀ ultramarathon runners recruited before two races | Questionnaire including items on DSs | Current use | 73 | 54 |
| Buxton & Hagan 2012[154] | 148 ♂ & 32 ♀ university athletes, Ghana | Questionnaire focused on energy drink consumption | One week prior to questionnaire | 72 | 42 |
| Hoyte et al. 2013[155] | 276 ♂ & 186 ♀ collegiate athletes from across the US, ~10 sports | Questionnaire focused on ergogenic DSs | Use in last year | Not specified | 63 |
| Blackhouse et al. 2013[176] | 138 ♂ & 74 ♀ competitive athletes, club/collegiate & national/international level, 32 sports | Questionnaire including items on DSs | Not clear | Not specified | 21 |
| Kim et al. 2013[203] | 70 ♂ & 31 ♀ Korean competitive judoists & 37 ♂ & 34 ♀ Japanese competitive judoists | Questionnaire focused on DSs | Use in last 6 months | 98 | 58 |
| Darvishi et al. 2013[156] | 173 ♂ Iranian university athletes | Questionnaire focused on DSs | Use in last 6 months | 90 | 54 |
| Aljaloud & Ibrahim 2013[68] | 105 ♂ Saudi Arabian professional soccer players | Questionnaire focused on DS use | Current use | Not specified | 21 |
| Giannopoulou et al. 2013[175] | 2013 ♂ & 832 ♀ Greek athletes, 33 sports | Questionnaire focused on DS use | Use in last month | 95 | 54 |
| Sajber et al. 2013[61] | 55 elite Croatian swimmers, national and international level | Questionnaire including items on DSs | Regular use | 99 | 46 |
| Sousa et al. 2013[14] | 200 ♂ & 92 ♀ elite Portuguese athletes, national level, 13 sports | Questionnaire focused on DS use | Use in last 12 months | Not specified | 50 |
| Wiens et al. 2014[177] | 231 ♂ & 335 ♀ young Canadian athletes (11-25 years) competing at club level, 20 sports | Questionnaire focused on DSs | Use in last 3 months | Not Specified | 50 |
| Bianco et al. 2014[169] | 434 ♂ & 127 ♀ performing resistance training in or near Palermo, Italy | Interview focused on protein in diet & protein supplements | Current use | Not specified | 46 |
| Sekulic et al. 2014[204] | 105 ♂ elite rugby union players | Questionnaire including items on DSs | Not clear | >99 | 75 |

Abbreviations: ♂=male, ♀=female, yr=year, HS=high school, US=United States, DS=dietary supplement

^a^Period over which investigators asked participants to report their dietary supplement use (e.g., current use, usual use, use in the last month, use in last year).

^b^Calculated as the sample providing the data divided by the total number of individuals who were asked to participate. In this calculation, the numerator included only the athletes whose data were used in the investigation. The values reported by some authors had to be recalculated because the authors reported the number of responses received without considering data that was discarded (e.g., due to incomplete or improperly completed questionnaires or food records).

^c^Based on method of Loney et al. [28], as described in the text

Electronic Supplementary Material Table S2. Prevalence of dietary supplement use by athletes^a^

| Study | Participants | % of Entire Sample Reporting Use | | | | | | | | | | | | | | | | | |
| --- | --- | --- | --- | --- | --- | --- | --- | --- | --- | --- | --- | --- | --- | --- | --- | --- | --- | --- | --- |
|  |  | Any DS use | Any vit (V), minl (M), or vit/  minl (VM) suppl | Multi-vit (with or wo minl) | Vit A | Vit B or B Cmpx | Vit C | Vit D | Vit E | Fe | Ca | Zn | AA  (A), Prot (P) | C  r  e  a  t  i  n  e | E  c  h  i  n  a  c  e  a | G  i  n  s  e  n  g | S  p  o  r  t  D  r  i  n  k | S  p  o  r  t  B  a  r | Other suppls in study |
| Bobb & Ryan 1969[116] | 12 basketball players, 10 ice hockey players, 6 swimmers |  |  | 25 |  |  |  |  |  |  |  |  |  |  |  |  |  |  |  |
| Steel 1970[139] | 66 ♂ & 14 ♀ Australian Oly athletes | 84 | VM-31 | 10 |  | 14 | 15 |  | 14 | 14 | 1 |  |  |  |  |  |  |  |  |
| Bright-See et al. 1978[83] | 51 ♀ ballet students | 88 |  |  |  |  |  |  |  |  |  |  |  |  |  |  |  |  |  |
| Werblow et al. 1978[146] | 94 ♀ collegiate athletes, 10 sports |  |  |  | 19 | 18 | 37 | 21 | 19 | 25 | 5 |  | P-7 |  |  |  | 4 |  |  |
| Houston 1980[54] | 8 elite ♂ Canadian swimmers | 75 | V-25 | 50 |  | 25 | 38 |  | 13 | 13 |  |  |  |  |  |  |  |  |  |
|  | 12 elite ♀ Canadian swimmers | 50 | V-17 | 42 |  | 13 | 25 |  | 0 | 25 |  |  |  |  |  |  |  |  |  |
| Barry et al. 1981[140] | 115 ♂ & 28 ♀ elite Irish athletes, 1980 Moscow Oly | 55 |  |  |  |  |  |  |  |  |  |  |  |  |  |  |  |  |  |
| Adams et al. 1982[55] | 19 ♀ college swimmers |  |  | 21 |  |  | 5 |  |  |  |  |  |  |  |  |  |  |  |  |
| Short & Short 1983[42] | 40 ♂collegiate football players |  |  | 43 |  | 15 | 30 | 13 | 8 |  |  |  |  |  |  |  |  |  |  |
|  | 38 ♂ collegiate wrestlers |  |  | 26 |  | 3 | 100 |  | 3 |  |  |  |  |  |  |  |  |  |  |
|  | 12 ♂ collegiate basketball players |  |  | 42 |  |  | 33 |  |  |  |  | 8 |  |  |  |  |  |  |  |
| Grandjean 1983[127] | 69 elite amateur & professional athletes |  | VM-92  V-38  M-20 |  |  | 4 |  |  |  |  |  |  | P-6 |  |  |  |  |  |  |
| Calabrese et al. 1983[84] | 25 ♀ professional ballet dancers | 40 |  | 12 |  |  |  |  |  |  |  |  |  |  |  |  |  |  |  |
| Moffatt 1984[90] | 13 ♀ elite HS female gymnasts | 23 |  | 8 |  |  | 15 |  |  |  |  |  |  |  |  |  |  |  |  |
| Douglas, 1984[157] | 515 ♂ & 425 ♀ HS athletes, 18 sports |  | V-19 |  |  |  |  |  |  |  |  |  |  |  |  |  |  |  |  |
| Parr et al. 1984[170] | 1,432♂ athletes |  | V-56  M-15 | 24 | 3 | 6 | 21 |  | 5 | 4 | 1 | 2 | P-12 |  |  |  |  |  |  |
|  | 1,547 ♀ athletes |  | V-33  M-10 |  |  |  |  |  |  |  |  |  | P-1 |  |  |  |  |  |  |
| Campbell & MacFadyen 1984[56] | 38 ♂ & 63♀ adolescent swimmers, (aged >13-20 yr) | 79 |  | 6 | 6 | 28 | 64 | 1 | 26 | 29 |  |  |  |  |  |  |  |  | Ω3FA -4 |
| Braisted et al. 1985[86] | 44 ♀ adolescent ballet dancers (aged 16±2 yr) | 56 |  | 34 |  |  | 36 |  |  | 5 | 2 |  |  |  |  |  |  |  |  |
| Grandjean 1985[128] | ~150 elite athletes, 12 sports | 52 | VM-39  V-22  M-12 |  |  |  |  |  |  |  |  |  | P-5 |  |  | 1 |  |  |  |
| Benson et al. 1985[87] | 92 adolescent ♀ ballet dancers (aged 12-17 yr) |  | VM-60 |  |  |  |  |  |  |  |  |  |  |  |  |  |  |  |  |
| Cohen et al. 1985[85] | 10 ♂ elite ballet dancers |  | V-81 |  |  |  |  |  |  |  |  |  |  |  |  |  |  |  |  |
|  | 12 ♀ elite ballet dancers |  | V-92 |  |  |  |  |  |  |  |  |  |  |  |  |  |  |  |  |
| Deuster et al. 1986[36, 37] & Singh et al. 1990[38] | 45-51 ♀ elite marathon runners |  | VM-53 |  |  |  |  |  |  |  |  | 21 |  |  |  |  |  |  | Cu-18 |
| Lampe et al. 1986[94] | 9 ♀marathon runners |  |  |  |  |  | 33 |  |  | 33 |  |  |  |  |  |  |  |  |  |
| Barr 1986[95] | 104 ♀ marathon runners | 75 |  | 42 |  | 21 | 41 |  | 15 | 38 | 19 |  |  |  |  |  |  |  | BY-8 |
|  | 105 ♀ fitness club members | 64 |  | 45 |  | 41 | 38 |  | 13 | 23 | 23 |  |  |  |  |  |  |  | BY-7 |
| Loosli et al. 1986[91] | 97 ♀adolescent gymnasts (aged11-17 yr) |  | VM-43 |  |  |  |  |  |  |  | 17 |  |  |  |  |  |  |  |  |
| Slavin et al. 1986[119] | 36 ♀elite cyclists |  |  | 64 |  | 56 | 58 |  |  | 47 | 11 |  | P-6 |  |  |  | 17 |  |  |
|  | 76 ♀ recreational cyclists |  |  | 20 |  | 18 | 32 |  |  | 21 | 13 |  | P-1 |  |  |  | 0 |  |  |
| Thibault et al. 1986[96] | 1,123 marathon runners |  |  | 20 |  |  |  |  |  |  |  |  |  |  |  |  |  |  |  |
| Khoo et al. 1987[98] | 19 ♂ triathletes |  | VM-60 |  |  |  |  |  |  |  |  |  |  |  |  |  |  |  |  |
|  | 10 ♀ triathletes |  | VM-80 |  |  |  |  |  |  |  |  |  |  |  |  |  |  |  |  |
| Barr 1987[147] | 70 ♀ collegiate athletes, 8 sports | 76 |  | 44 |  | 10 | 36 |  | 1 | 31 | 9 |  |  |  |  |  |  |  |  |
| Evers 1987[103] | 21 ♀ dance students | 48 |  |  |  |  |  |  |  | 29 |  |  |  |  |  |  |  |  |  |
| Faber 1987[69] | 74 ♂ body-builders | 63 |  | 37 | 10 | 14 | 31 |  | 22 |  |  |  | P-59 |  |  |  |  |  | DL-20;Dol-18; BY-15;  Kelp-11; PE-7 |
| Burke & Read 1988[62] | 54 ♂ elite soccer players |  | VM-48 |  |  |  |  |  |  |  |  |  |  |  |  |  |  |  |  |
| Schulz 1988[79] | 66 ♂ & 61 ♀ basketball players & X-C runners | 44 |  | 30 |  |  | 15 |  |  | 11 | 11 |  |  |  |  |  |  |  |  |
|  | 24 ♂ & 66 ♀ exercisers | 46 |  | 15 |  |  | 10 |  |  | 5 | 6 |  |  |  |  |  |  |  |  |
| Clark et al. 1988[110] | 93 ♀elite middle- & long-distance runners | 71 |  | 50 |  | 25 | 38 |  |  | 67 | 33 |  |  |  |  |  |  |  |  |
| Nowak et al. 1988[78] | 16 ♂ collegiate basketball players | 6 |  |  |  |  |  |  |  |  |  |  |  |  |  |  |  |  |  |
|  | 10 ♀ collegiate basketball players | 50 |  |  |  |  |  |  |  |  |  |  |  |  |  |  |  |  |  |
| Lamar-Hildebrand et al. 1989[72] | 10 ♀ bodybuilders |  | VM-80 |  |  |  |  |  |  |  |  |  | A-40 |  |  |  |  |  |  |
| Krowchuk et al. 1989[158] | 212 ♂ HS athletes | 44 | V-33 |  |  |  |  |  |  |  |  |  | 35 |  |  |  |  |  |  |
|  | 83 ♀HS athletes | 30 |  |  |  |  |  |  |  |  |  |  |  |  |  |  |  |  |  |
| Snyder et al. 1989[122] | 10 ♂ elite speed skaters | 60^m^ |  |  |  |  |  |  |  |  |  |  |  |  |  |  | 90 |  |  |
|  | 7 ♀elite speed skaters | 86 ^m^ |  |  |  |  |  |  |  | 43 | 14 |  |  |  |  |  | 71 |  |  |
| Sandoval et al. 1989[70] | 5 ♂ body builders |  |  | 20 |  |  |  |  |  |  |  |  | AP-60 |  |  |  |  |  |  |
|  | 6 ♀ body builders |  |  | 50 |  |  |  |  |  |  |  |  | AP-67 |  |  |  |  |  |  |
| Neiman et al. 1989[97] | 291♂ marathon runners | 30 |  | 22 | 2 | 7 | 14 | 1 | 6 | 2 | 7 | 5 |  |  |  |  |  |  |  |
|  | 56 ♀ marathon runners | 27 |  | 20 | 2 | 5 | 7 | 2 | 7 | 2 | 7 | 0 |  |  |  |  |  |  |  |
| Heyward et al. 1989[71] | 7♂ bodybuilders |  |  |  |  |  |  |  |  |  |  |  | A-29  P-0 |  |  |  |  |  |  |
|  | 12 ♀ bodybuilders |  |  |  |  |  |  |  |  |  |  |  | A-33  P-25 |  |  |  |  |  |  |
| Worme et al. 1990[99] | 50 ♂ triathletes |  |  | 30 |  |  |  |  |  |  |  |  |  |  |  |  |  |  |  |
|  | 21 ♀ triathletes |  |  | 57 |  |  |  |  |  |  |  |  |  |  |  |  |  |  |  |
| Frusztajer et al. 1990[88] | 20 ♀classical ballet dancers |  | VM-65  V-60 |  |  |  |  |  |  |  |  |  |  |  |  |  |  |  |  |
| Kleiner et al. 1990[73] | 19 ♂ elite bodybuilders | 90 |  |  |  |  |  |  |  |  |  |  |  |  |  |  |  |  |  |
|  | 8 ♀ elite bodybuilders | 100 |  |  |  |  |  |  |  |  |  |  |  |  |  |  |  |  |  |
| Pate et al. 1990[111] | 103 ♀ runners |  |  | 46 |  |  | 46 |  |  | 50 | 49 |  |  |  |  |  |  |  |  |
| Ersoy 1991[92] | 20 ♀ Turkish gymnasts, aged 12±1 yrs |  | VM-45 |  |  |  |  |  |  |  |  |  |  |  |  |  |  |  |  |
| Burke et al. 1991[63] | 25 ♂elite Australian triathletes | 44 |  |  |  |  |  |  |  |  |  |  |  |  |  |  |  |  |  |
|  | 19 ♂elite Australian marathon runners | 68 |  |  |  |  |  |  |  |  |  |  |  |  |  |  |  |  |  |
|  | 56 ♂elite Australian soccer players |  | V-14 |  |  |  |  |  |  |  |  |  |  |  |  |  |  |  |  |
|  | 19 ♂elite Australian Oly weightlifters | 100 |  | 84 |  | 37 | 63 |  | 32 |  | 63 |  | P-74  A-11 |  |  |  |  |  | Comfrey-37 |
| Faber & Benade 1991[125] | 20 ♂ elite male field athletes |  | VM-35 |  |  |  |  |  |  |  |  |  |  |  |  |  |  |  |  |
|  | 10 ♀ elite field athletes |  | VM-33 |  |  |  |  |  |  |  |  |  |  |  |  |  |  |  |  |
| Hawley & Williams 1991[57] | 9 ♂ swimmers, aged 13±1 yr |  |  | 33 |  |  |  |  |  |  |  |  |  |  |  |  |  |  |  |
|  | 11 ♀ swimmers, aged 13±2 yr |  |  | 9 |  |  |  |  |  |  |  |  |  |  |  |  |  |  |  |
| Soper et al. 1992[118] | 535 aerobic dance instructors | 51 | V-46  M-24 |  |  |  |  |  |  |  |  |  | AP-8 |  |  |  |  |  |  |
| Frederick & Hawkins 1992 [104] | 14 ♀ collegiate dancers |  | VM-29 |  |  |  |  |  |  |  | 0 |  |  |  |  |  |  |  |  |
|  | 13 ♀ collegiate track team members |  | VM-39 |  |  |  |  |  |  |  | 0 |  |  |  |  |  |  |  |  |
| Stensland & Sobal 1992[201] | 17 ♂ dancers | 65 |  | 51 |  | 5 | 16 |  | 5 | 5 | 7 |  |  |  |  |  |  |  |  |
|  | 89 ♀ dancers | 59 |  |  |  |  |  |  |  |  |  |  |  |  |  |  |  |  |  |
| Fogelholm et al. 1992[129] | 427 ♂ elite Finnish athletes, 11 sports^f^ |  |  |  |  | 34-60 |  |  |  | 31-57 |  |  | AP37-59 |  |  |  |  |  | Mg&Zn-24-49 |
| Jacobson & Aldana 1992[43] | 533 ♂ collegiate athletes, 8 sports |  | V-40 |  |  |  |  |  |  |  |  |  | P-39  A-23 |  |  |  |  |  |  |
|  | 259 ♀ collegiate athletes, 6 sports |  | V-35 |  |  |  |  |  |  |  |  |  | P-19  A-3 |  |  |  |  |  |  |
|  | Collegiate football players |  | V-50 |  |  |  |  |  |  |  |  |  | P-42  A-25 |  |  |  |  |  |  |
|  | Collegiate basketball players |  | V-35 |  |  |  |  |  |  |  |  |  | P-21  A-7 |  |  |  |  |  |  |
|  | Collegiate wrestlers |  | V-60 |  |  |  |  |  |  |  |  |  | P-33  A-33 |  |  |  |  |  |  |
|  | Collegiate softball players |  | V-19 |  |  |  |  |  |  |  |  |  | P-27  A-5 |  |  |  |  |  |  |
|  | Collegiate baseball players |  | V-30 |  |  |  |  |  |  |  |  |  | P-31  A-23 |  |  |  |  |  |  |
|  | Collegiate track athletes |  | V-54 |  |  |  |  |  |  |  |  |  | P-26  A-9 |  |  |  |  |  |  |
|  | Collegiate tennis players |  | V-33 |  |  |  |  |  |  |  |  |  | P-25  A-8 |  |  |  |  |  |  |
|  | Collegiate volleyball players |  | V-20 |  |  |  |  |  |  |  |  |  | P-30  A-0 |  |  |  |  |  |  |
| Singh et al. 1993[106] | 15 ♂ & 2 ♀ ultra-marathoners | 76 |  |  |  | 53 | 59 |  | 59 | 53 |  |  |  |  |  |  |  |  |  |
| Bazzarre et al. 1993[171] | 61 ♀ recreational athletes |  | VM-51 | 43 |  | 8 | 11 |  | 4 | 15 | 3 |  | P-8 |  |  |  |  |  |  |
|  | 30 ♀ recreational athletes |  | VM-50 |  |  |  |  |  |  |  |  |  |  |  |  |  |  |  |  |
| Oppliger et al. 1993[82] | 713 ♂ HS wrestlers |  |  | 40 |  | 32 | 50 |  | 28 |  |  |  | P-30  A-25 |  |  |  |  |  |  |
| Walberg-Rankin et al. 1993[75] | 6 ♀ bodybuilders | 100 |  | 33 |  | 33 | 33 |  | 17 | 17 | 17 |  | P-50  A-100 |  |  |  |  |  | Car-33 |
| Linseisen et al. 1993[74] | 13 ♂ German bodybuilders | 100 | VM-100 |  |  |  |  |  |  |  |  |  | PA-100 |  |  |  |  |  |  |
| Sobal 1994[44] | 430 ♂ & 312 ♀ HS athletes, >15 sports | 38 |  | 18 | 9 | 8 | 25 | 5 | 7 | 11 | 9 | 3 |  |  |  |  |  |  |  |
|  | 133 track & field athletes |  | VM-31 |  |  |  |  |  |  |  |  |  |  |  |  |  |  |  |  |
|  | 113 baseball/softball players |  | VM-34/50 |  |  |  |  |  |  |  |  |  |  |  |  |  |  |  |  |
|  | 91 basketball players |  | VM-40 |  |  |  |  |  |  |  |  |  |  |  |  |  |  |  |  |
|  | 83 football players |  | VM-39 |  |  |  |  |  |  |  |  |  |  |  |  |  |  |  |  |
|  | 72 soccer players |  | VM-33 |  |  |  |  |  |  |  |  |  |  |  |  |  |  |  |  |
|  | 44 wrestlers |  | VM-59 |  |  |  |  |  |  |  |  |  |  |  |  |  |  |  |  |
|  | 36 lacrosse players |  | VM-25 |  |  |  |  |  |  |  |  |  |  |  |  |  |  |  |  |
|  | 36 X-C runners |  | VM-47 |  |  |  |  |  |  |  |  |  |  |  |  |  |  |  |  |
|  | 23 volleyball players |  | VM-44 |  |  |  |  |  |  |  |  |  |  |  |  |  |  |  |  |
|  | 19 tennis players |  | VM-32 |  |  |  |  |  |  |  |  |  |  |  |  |  |  |  |  |
|  | 10 ice hockey players |  | VM-50 |  |  |  |  |  |  |  |  |  |  |  |  |  |  |  |  |
|  | 10 gymnasts |  | VM-40 |  |  |  |  |  |  |  |  |  |  |  |  |  |  |  |  |
|  | 7 skiers |  | VM-15 |  |  |  |  |  |  |  |  |  |  |  |  |  |  |  |  |
|  | 4 swimmers |  | VM-25 |  |  |  |  |  |  |  |  |  |  |  |  |  |  |  |  |
|  | 4 golfers |  | VM-50 |  |  |  |  |  |  |  |  |  |  |  |  |  |  |  |  |
| Musaiger 1994[130] | 304 elite athletes in Bahrain, 4 sports | 4^n^ |  |  |  |  |  |  |  |  |  |  |  |  |  |  | 8 |  |  |
| Brill & Keane 1994[6] | 210 ♂ bodybuilders |  | V-70  M-49 |  |  |  |  |  |  |  |  |  | P-61  A-51 |  |  |  | 31 | 8 |  |
|  | 99 ♀ bodybuilders |  | V-76  M-57 |  |  |  |  |  |  |  |  |  | P-54  A-59 |  |  |  | 38 | 17 |  |
| Massad, 1995[159] | 302 ♂ & 205 ♀ HS athletes, ~34 sports |  |  | 42 | 13 | 13 | 30 |  | 13 | 15 | 18 | 7 | P-22  A-9 |  |  | 5 | 43 |  | Se-5;BP-5 |
| Andersen et al. 1995[76] | 49 ♂ body builders |  |  | 51 |  | 28 | 46 | 32 |  |  | 33 |  | P-38  A-58 |  |  |  |  |  | K-30 |
| Peters & Goetzsche 1997[107] | 150 ♂ ultramarathoners | 75 | VM-62 |  |  |  |  |  |  |  |  |  |  |  |  |  |  |  | Mg-70 |
|  | 23 ♀ ultramarathoners | 83 | VM-78 |  |  |  |  |  |  | 52 |  |  |  |  |  |  |  |  | Mg-57 |
| Beals & Manore 1998[172] | 48 ♀ athletes, 9 sports |  | VM-46 |  |  |  |  |  |  |  |  |  |  |  |  |  |  |  |  |
| Ziegler et al. 1998[113] | 12 ♂ & 16 ♀ adolescent figure skaters (aged ~15±2 yr) | 0 |  |  |  |  |  |  |  |  |  |  |  |  |  |  | 0 |  |  |
| Jonnalagadda et al. 1998[93] | 33 ♀elite artistic gymnasts |  | VM-92 |  |  |  |  |  |  |  |  |  |  |  |  |  |  |  |  |
| Felder et al. 1998[123] | 10 ♀ professional surfers | 50 |  | 40 |  |  | 40 |  |  |  |  |  |  |  | 60* |  | 20 |  |  |
| Rosen 1999[115] | 33 ♂ elite power athletes | 88 | M-62 | 74 |  |  | 45 |  |  | 28 |  |  | AP-18 | 28 |  |  |  |  | Ω3FA-24 |
|  | 19 ♂ elite alpine skiers | 70 | M-30 | 48 |  |  | 10 |  |  | 37 |  |  | AP-0 | 11 |  |  |  |  | Ω3FA-10 |
|  | 14 ♀ elite alpine skiers |  |  | 28 |  |  | 14 |  |  | 7 |  |  | AP-0 | 8 |  |  |  |  | Ω3FA-14 |
|  | 17 ♂ elite X-C skiers | 95 | M-32 | 35 |  |  | 47 |  |  | 18 |  |  | AP-0 | 0 |  |  |  |  |  |
|  | 17 ♀ elite X-C skiers |  |  | 24 |  |  | 24 |  |  | 24 |  |  | AP-0 | 0 |  |  |  |  | Ω3FA-53 |
| LaBotz & Smith 1999[45] | 739 ♂ & ♀collegiate athletes |  |  |  |  |  |  |  |  |  |  |  | AP-4 |  |  |  |  |  |  |
|  | ♂ baseball players |  |  |  |  |  |  |  |  |  |  |  |  | 81 |  |  |  |  |  |
|  | ♂ basketball players |  |  |  |  |  |  |  |  |  |  |  |  | 35 |  |  |  |  |  |
|  | ♀ basketball players |  |  |  |  |  |  |  |  |  |  |  |  | 14 |  |  |  |  |  |
|  | ♂ cheerleaders |  |  |  |  |  |  |  |  |  |  |  |  | 44 |  |  |  |  |  |
|  | ♀ cheerleaders |  |  |  |  |  |  |  |  |  |  |  |  | 0 |  |  |  |  |  |
|  | ♂divers |  |  |  |  |  |  |  |  |  |  |  |  | 67 |  |  |  |  |  |
|  | ♀ divers |  |  |  |  |  |  |  |  |  |  |  |  | 0 |  |  |  |  |  |
|  | ♂ football players |  |  |  |  |  |  |  |  |  |  |  |  | 71 |  |  |  |  |  |
|  | ♂fencers |  |  |  |  |  |  |  |  |  |  |  |  | 0 |  |  |  |  |  |
|  | ♀ fencers |  |  |  |  |  |  |  |  |  |  |  |  | 0 |  |  |  |  |  |
|  | ♀ field hockey players |  |  |  |  |  |  |  |  |  |  |  |  | 0 |  |  |  |  |  |
|  | ♂ golfers |  |  |  |  |  |  |  |  |  |  |  |  | 0 |  |  |  |  |  |
|  | ♀ golfers |  |  |  |  |  |  |  |  |  |  |  |  | 0 |  |  |  |  |  |
|  | ♀ gymnasts |  |  |  |  |  |  |  |  |  |  |  |  | 0 |  |  |  |  |  |
|  | ♂ lacrosse players |  |  |  |  |  |  |  |  |  |  |  |  | 30 |  |  |  |  |  |
|  | ♀ lacrosse players |  |  |  |  |  |  |  |  |  |  |  |  | 0 |  |  |  |  |  |
|  | ♀ rowers (crew) |  |  |  |  |  |  |  |  |  |  |  |  | 0 |  |  |  |  |  |
|  | ♂ soccer players |  |  |  |  |  |  |  |  |  |  |  |  | 33 |  |  |  |  |  |
|  | ♀ soccer players |  |  |  |  |  |  |  |  |  |  |  |  | 0 |  |  |  |  |  |
|  | ♂ swimmers |  |  |  |  |  |  |  |  |  |  |  |  | 52 |  |  |  |  |  |
|  | ♀ swimmers |  |  |  |  |  |  |  |  |  |  |  |  | 19 |  |  |  |  |  |
|  | ♂ tennis players |  |  |  |  |  |  |  |  |  |  |  |  | 50 |  |  |  |  |  |
|  | ♀ tennis players |  |  |  |  |  |  |  |  |  |  |  |  | 13 |  |  |  |  |  |
|  | ♂track/field/X-C |  |  |  |  |  |  |  |  |  |  |  |  | 35 |  |  |  |  |  |
|  | ♀ track/field/X-C |  |  |  |  |  |  |  |  |  |  |  |  | 10 |  |  |  |  |  |
|  | ♀ volleyball players |  |  |  |  |  |  |  |  |  |  |  |  | 0 |  |  |  |  |  |
|  | ♂ wrestlers |  |  |  |  |  |  |  |  |  |  |  |  | 44 |  |  |  |  |  |
| Krumbach et al. 1999[7] | 266 ♂ & 145 ♀ collegiate athletes |  | VM-57 |  |  |  |  |  |  |  |  |  |  |  |  |  |  |  |  |
|  | 7 ♂ tennis players |  | VM-83 | 74 | 6 | 13 | 36 |  | 8 | 3 | 8 | 7 |  |  |  |  |  |  | Vit B_12_-6 |
|  | 16 ♂ gymnasts |  | VM-81 |  |  |  |  |  |  |  |  |  |  |  |  |  |  |  |  |
|  | 21♂ track&field athletes |  | VM-71 |  |  |  |  |  |  |  |  |  |  |  |  |  |  |  |  |
|  | 28 ♂ baseball players |  | VM-64 |  |  |  |  |  |  |  |  |  |  |  |  |  |  |  |  |
|  | 18 ♂ swimmers/divers |  | VM-67 |  |  |  |  |  |  |  |  |  |  |  |  |  |  |  |  |
|  | 12 ♂ X-C runners |  | VM-58 |  |  |  |  |  |  |  |  |  |  |  |  |  |  |  |  |
|  | 126 ♂ football players |  | VM-52 |  |  |  |  |  |  |  |  |  |  |  |  |  |  |  |  |
|  | 11 ♂ cheer/dancers |  | VM-45 |  |  |  |  |  |  |  |  |  |  |  |  |  |  |  |  |
|  | 7 ♂ wrestlers |  | VM-29 |  |  |  |  |  |  |  |  |  |  |  |  |  |  |  |  |
|  | 11 ♂ golfers |  | VM-27 |  |  |  |  |  |  |  |  |  |  |  |  |  |  |  |  |
|  | 10 ♂ basketball players |  | VM-20 |  |  |  |  |  |  |  |  |  |  |  |  |  |  |  |  |
|  | 7 ♀ X-C runners |  | VM-86 | 72 | 0 | 6 | 40 |  | 4 | 17 | 18 | 7 |  |  |  |  |  |  | Vit B_12_-12 |
|  | 11 ♀ gymnasts |  | VM-82 |  |  |  |  |  |  |  |  |  |  |  |  |  |  |  |  |
|  | 5 ♀ tennis players |  | VM-80 |  |  |  |  |  |  |  |  |  |  |  |  |  |  |  |  |
|  | 23 ♀ swimmers/divers |  | VM-78 |  |  |  |  |  |  |  |  |  |  |  |  |  |  |  |  |
|  | 11 ♀ golfers |  | VM-64 |  |  |  |  |  |  |  |  |  |  |  |  |  |  |  |  |
|  | 17 ♀ soccer players |  | VM-59 |  |  |  |  |  |  |  |  |  |  |  |  |  |  |  |  |
|  | 9 ♀ volleyball players |  | VM-56 |  |  |  |  |  |  |  |  |  |  |  |  |  |  |  |  |
|  | 10 ♀ cheer/dancers |  | VM-50 |  |  |  |  |  |  |  |  |  |  |  |  |  |  |  |  |
|  | 25♀ track&field athletes |  | VM-48 |  |  |  |  |  |  |  |  |  |  |  |  |  |  |  |  |
|  | 14 ♀ softball players |  | VM-43 |  |  |  |  |  |  |  |  |  |  |  |  |  |  |  |  |
|  | 13 ♀ basketball players |  | VM-31 |  |  |  |  |  |  |  |  |  |  |  |  |  |  |  |  |
| Kim & Keen 1999[160] | 926 ♂ & 429 ♀ HS athletes, South Korea, >15 sports |  | VM-36 |  | 2 | 22 | 23 |  | 13 | 4 | 4 | 1 |  |  |  |  |  |  | Vit B_1_-11  Vit B_2_-22  Vit B_3_-5  Vit B_5_-11  Vit B_6_-22  Vit B_9_-4  Vit B_12_-12 |
| Swirzinski  2000[8] | 170 ♂ HS football players | 31 | V-4 |  |  |  |  |  |  |  |  |  | A-1 | 29 |  |  |  |  |  |
| Greenwood et al. 2000[46] | 156 ♂ & 63 ♀ collegiate athletes |  |  |  |  |  |  |  |  |  |  |  | A-8^v^ |  |  |  | 9 |  |  |
|  | 76 ♂ football players |  |  |  |  |  |  |  |  |  |  |  |  | 67 |  |  |  |  |  |
|  | 33 ♂ baseball players |  |  |  |  |  |  |  |  |  |  |  |  | 45 |  |  |  |  |  |
|  | 27 ♂track & field |  |  |  |  |  |  |  |  |  |  |  |  | 56 |  |  |  |  |  |
|  | 14 ♂ basketball players |  |  |  |  |  |  |  |  |  |  |  |  | 36 |  |  |  |  |  |
|  | 8 ♂ golfers |  |  |  |  |  |  |  |  |  |  |  |  | 26 |  |  |  |  |  |
|  | 20 ♀ track & field |  |  |  |  |  |  |  |  |  |  |  |  | 1 |  |  |  |  |  |
|  | 4 ♀ tennis players |  |  |  |  |  |  |  |  |  |  |  |  | 25 |  |  |  |  |  |
|  | ♀ basketball players |  |  |  |  |  |  |  |  |  |  |  |  | 0 |  |  |  |  |  |
|  | ♀ volleyball players |  |  |  |  |  |  |  |  |  |  |  |  | 0 |  |  |  |  |  |
|  | ♀ golfers |  |  |  |  |  |  |  |  |  |  |  |  | 0 |  |  |  |  |  |
| Smith & Dahm 2000[47] | 182 ♂ HS athletes |  |  |  |  |  |  |  |  |  |  |  |  | 14 |  |  |  |  |  |
|  | 146 ♀ HS athletes |  |  |  |  |  |  |  |  |  |  |  |  | 1 |  |  |  |  |  |
|  | 73 ♂ football players |  |  |  |  |  |  |  |  |  |  |  |  | 29 |  |  |  |  |  |
|  | 70 soccer players |  |  |  |  |  |  |  |  |  |  |  |  | 3 |  |  |  |  |  |
|  | 22 hockey players |  |  |  |  |  |  |  |  |  |  |  |  | 14 |  |  |  |  |  |
|  | 28 volleyball players |  |  |  |  |  |  |  |  |  |  |  |  | 4 |  |  |  |  |  |
|  | 9 dancers |  |  |  |  |  |  |  |  |  |  |  |  | 0 |  |  |  |  |  |
|  | 8 tennis players |  |  |  |  |  |  |  |  |  |  |  |  | 0 |  |  |  |  |  |
|  | 5 golfers |  |  |  |  |  |  |  |  |  |  |  |  | 0 |  |  |  |  |  |
|  | 4 track & X-C |  |  |  |  |  |  |  |  |  |  |  |  | 0 |  |  |  |  |  |
|  | 1 ♂ wrestler |  |  |  |  |  |  |  |  |  |  |  |  | 0 |  |  |  |  |  |
| Sheppard et al. 2000[163] | 85 ♂ & 11 ♀ members of health clubs |  | V-78  M-60 |  |  |  |  |  |  |  |  |  | P-67 | 57 |  |  |  |  | Caf-48 |
| Kanayama et al. 2001[164] | 334 ♂ gym users |  |  |  |  |  |  |  |  |  |  |  | P-27 | 11 |  |  |  |  |  |
|  | 177 ♀ gym users |  |  |  |  |  |  |  |  |  |  |  | P-11 | 1 |  |  |  |  |  |
| Jacobson et al. 2001[148] | 205 ♂ collegiate athletes, 11 sports | 79 | VM-11 |  |  |  |  |  |  |  |  |  | P-10  A-2 | 32 |  |  | 20^d^ | 5 |  |
|  | 125 ♀ collegiate athletes, 8 sports, | 65 | VM-22 |  |  |  |  |  |  |  |  |  | P-5  A-2 | 7 |  |  | 18^d^ | 12 |  |
| Jonnalagadda 2001[50] | 31 ♂ collegiate freshman football players | 42 | V-23 |  |  |  |  |  |  |  |  |  | P-16  A-3 | 36 |  |  |  | 3 |  |
| Berglund 2001[141] | 116 ♂ Swedish athletes in Atlanta Oly Games (1996) | 42 | V-27  M-30 |  |  |  |  |  |  |  |  |  | A-6 | 16 |  | 14 |  |  | Ω3FA-8 |
|  | 67 ♀ Swedish athletes in Atlanta Oly Games (1996) | 57 | V-50  M-36 |  |  |  |  |  |  |  |  |  | A-12 | 3 |  | 12 |  |  | Ω3FA-5 |
|  | 56 ♂ Swedish athletes in Nagano Oly Games (1996) | 16 | V-13  M-13 |  |  |  |  |  |  |  |  |  | A-2 | 2 |  | 2 |  |  | Ω3FA-17 |
|  | 49 ♀ Swedish athletes in Nagano Oly Games (1996) | 35 | V-33  M-22 |  |  |  |  |  |  |  |  |  | A-0 | 0 |  | 2 |  |  | Ω3FA-20 |
|  | 99 ♂ Swedish athletes in Sydney Oly Games (1996) | 19 | V-10  M-9 |  |  |  |  |  |  |  |  |  | A-2 | 7 |  | 3 |  |  |  |
|  | 58 ♀ Swedish athletes in Sydney Oly Games (1996) | 31 | V-21  M-22 |  |  |  |  |  |  |  |  |  | A-5 | 2 |  | 5 |  |  |  |
| Ray et al. 2001[161] | 469 ♀ adolescent athletes (aged 13-19 yr) |  |  |  |  |  |  |  |  |  |  |  |  | 23 |  |  |  |  |  |
|  | 205 ♀ adolescent athletes (aged 13-19 yr) |  |  |  |  |  |  |  |  |  |  |  |  | 2 |  |  |  |  |  |
| Metzl et al. 2001[48] | 604 ♂ middle & HS athletes |  |  |  |  |  |  |  |  |  |  |  |  | 9 |  |  |  |  |  |
|  | 492 ♀ middle & HS athletes |  |  |  |  |  |  |  |  |  |  |  |  | 2 |  |  |  |  |  |
|  | 11 gymnasts |  |  |  |  |  |  |  |  |  |  |  |  | 36 |  |  |  |  |  |
|  | 40 hockey players |  |  |  |  |  |  |  |  |  |  |  |  | 20 |  |  |  |  |  |
|  | 56 wrestlers |  |  |  |  |  |  |  |  |  |  |  |  | 14 |  |  |  |  |  |
|  | 179 football players |  |  |  |  |  |  |  |  |  |  |  |  | 13 |  |  |  |  |  |
|  | 143 lacrosse players |  |  |  |  |  |  |  |  |  |  |  |  | 13 |  |  |  |  |  |
|  | 40 cheerleaders |  |  |  |  |  |  |  |  |  |  |  |  | 8 |  |  |  |  |  |
|  | 61 swimmers |  |  |  |  |  |  |  |  |  |  |  |  | 7 |  |  |  |  |  |
|  | 52 skiers |  |  |  |  |  |  |  |  |  |  |  |  | 6 |  |  |  |  |  |
|  | 91 tennis players |  |  |  |  |  |  |  |  |  |  |  |  | 4 |  |  |  |  |  |
|  | 162 baseball players |  |  |  |  |  |  |  |  |  |  |  |  | 4 |  |  |  |  |  |
|  | 256 soccer players |  |  |  |  |  |  |  |  |  |  |  |  | 4 |  |  |  |  |  |
|  | 117 field hockey players |  |  |  |  |  |  |  |  |  |  |  |  | 2 |  |  |  |  |  |
|  | 280 track athletes |  |  |  |  |  |  |  |  |  |  |  |  | 1 |  |  |  |  |  |
|  | 527 weight trainers |  |  |  |  |  |  |  |  |  |  |  |  | 10 |  |  |  |  |  |
| Baylis et al. 2001[58] | 40 ♂ & 37 ♀ elite Australian swimmers | 99 | VM-95 | 58 |  | 22 | 73 |  | 31 | 30 | 5 | 26 | AP-18 | 31 | 39 | 8 | 87 | 8 | Mg-18  Inosine-16  CoQ-7  Gar/HR-16 |
| Mason et al. 2001[49] | 495 ♂ HS football players | 8 |  | 1 |  |  |  |  |  |  | <1 |  | A-3 | 6 |  | 1 |  |  |  |
|  | 407 ♀ HS volleyball players | 2 |  | 1 |  |  |  |  |  |  | 1 |  | A-<1 | <1 | 1 | 0 |  |  |  |
| Mullins et al. 2001[126] | 17 ♀ elite heptathletes |  | V-53  M-24 |  |  |  |  |  |  |  |  |  | P-24 |  |  |  |  |  |  |
| Ziegler et al. 2002[114] | 18 ♀ young figure skaters (aged 14-16 yr) | 30 |  |  |  |  |  |  |  |  |  |  |  |  |  |  |  |  |  |
| Schroder et al. 2002[80] | 55 ♂ elite Spanish basketball players | 58 |  | 49 |  |  | 15 |  |  | 7 |  |  | A-15  P-13 |  |  | 6 | 22 |  | Car-11;  Inosine-6;  Lecithin-6;  CoQ-4 |
| Beals 2002[81] | 23 ♀ elite adolescent volleyball players (aged 16±1 yr) |  | VM-26 |  |  |  |  |  |  |  |  |  |  |  |  |  |  |  |  |
| Sundgot-Borgen et al. 2003[9] | 666 ♂ elite Norwegian athletes | 51 | V-36  M-13 |  |  |  |  |  |  |  |  |  | A-6 | 6 |  | 5 |  |  | Ω3FA -18 |
|  | 556 ♀ elite Norwegian athletes | 54 | V-44  M-23 |  |  |  |  |  |  |  |  |  | A-2 | 2 |  | 3 |  |  | Ω3FA -20 |
| Ziegler et al. 2003[15] | 46 ♂ elite figure skaters | 65 |  | 61 |  |  |  |  |  |  |  |  | P-15  A-8 | 3 | 28 | 20 | 33 | 38 | GB-8; Gar-5  GT-5; KK-5;  Cham-8 |
|  | 59 ♀ elite figure skaters | 76 |  | 83* |  |  |  |  |  |  |  |  | P-11  A-2 | 0 | 44 | 10 | 35 | 27 |  |
| Ama et al. 2003[64] | 1,037 ♂ soccer players in Cameroon |  |  |  |  |  | 80 |  |  |  |  |  |  |  |  |  |  |  |  |
|  | 79 ♀ soccer players in Cameroon |  |  |  |  |  | 68 |  |  |  |  |  |  |  |  |  |  |  |  |
| Slater et al. 2003[131] | 85 ♂ elite Singaporean athletes, ~30 sports | 77 |  | 21 |  |  | 33 |  |  |  |  |  | P-13 | 16 |  | 15 | 39 | 15 | EC-19  BN-14  Caf-37 |
|  | 75 ♀ elite Singaporean athletes, ~30 sports | 81 |  |  |  |  |  |  |  |  |  |  |  |  |  |  |  |  |  |
| Corrigan & Kazlauskas 2003[142] | 2,758 athletes, Sydney Oly (2000), 32 sports |  | V-51  M-21 | 41 |  |  |  |  |  |  |  |  | A-13 |  |  |  |  |  |  |
| Herbold 2004[149] | 162 ♀ collegiate athletes, 9 sports | 65 |  | 40 | 6 | 7 | 32 | 5 | 9 | 8 | 14 | 3 | AP-12 |  | 14 | 6 | 73 | 50 | Cr-4; GS-4; WG-6 |
| Beitz et al. 2004[173] | 1,015 ♂ Germans engaged in sport |  | VM-32 |  |  |  |  |  |  |  |  |  |  |  |  |  |  |  |  |
|  | 1,271 ♀ Germans engaged in sport |  | VM-33 |  |  |  |  |  |  |  |  |  |  |  |  |  |  |  |  |
| Nogueira & DaCosta 2004[100] | 29 ♂ Brazilian triathletes | 62 |  |  |  |  |  |  |  |  |  |  | P-41 | 3 |  |  |  |  |  |
|  | 9 ♀ Brazilian triathletes | 33 |  |  |  |  |  |  |  |  |  |  | P-11 | 0 |  |  |  |  |  |
| Morrison 2004[10] | 182 ♂ & 40 ♀ using gyms in Long Island, New York | 85 |  | 54 |  | 16 | 35 |  | 23 | 10 | 17 |  | P-42  A-24^w^ | 13 |  | 4 |  | 42 | CP-8 |
| Froiland 2004[11] | 115 ♂ collegiate athletes, ~15 sports | 61 | V-33  M-18 | 22 | 2 | 3 | 18 | 3 | 9 | 3 | 7 | 4 | P-41  A-13^o^ | 35 | 4 | 10 | 48 |  |  |
|  | 88 ♀ collegiate athletes, ~15 sports |  | V-34  M-21 | 26 | 1 | 3 | 14 | 5 | 6 | 8 | 12 | 4 | P-8  A-2^o^ | 3 | 5 | 3 | 27 |  |  |
| Burns 2004[150] | 118 ♂ & 118 ♀ collegiate athletes,16 sports | 88 | VM-73 |  |  |  |  |  |  |  |  |  | P-40 | 31 |  |  |  |  |  |
| Paschoal & Amancio 2004[59] | 8 ♂ elite Brazilian swimmers |  | VM-13 |  |  |  | 13 |  |  |  |  |  |  |  |  |  |  |  |  |
| Bartee et al. 2004[162] | 1,014 ♂ HS athletes | 29 |  |  |  |  |  |  |  |  |  |  |  |  |  |  |  |  |  |
|  | 721 ♀ HS athletes | 18 |  |  |  |  |  |  |  |  |  |  |  |  |  |  |  |  |  |
| Nieper 2005[16] | 20 ♂ elite track & field athletes, UK | 55 |  |  |  |  |  |  |  |  |  |  |  |  |  |  |  |  |  |
|  | 12 ♀ elite track & field athletes, UK | 75 |  |  |  |  |  |  |  |  |  |  |  |  |  |  |  |  |  |
| Waddington et al. 2005[65] | 706 ♂ professional UK soccer players |  | V-58  M-23 |  |  |  |  |  |  |  |  |  | P-24 | 37 |  |  |  |  |  |
| Kristiansen et al. 2005[151] | 120 ♂ collegiate athletes, 10 sports, Canada |  | VM-52 | 52 |  |  |  |  |  | 3 |  |  | P-36 | 9 |  |  | 87 | 65 |  |
|  | 89 ♀ collegiate athletes, 10 sports, Canada |  | VM-63 | 63 |  |  |  |  |  | 25 |  |  | P-20 | 0 |  |  | 64 | 64 |  |
| Cole et al. 2005[51] | 28 ♂ collegiate football players |  | V-4 |  |  |  |  |  |  |  |  |  |  |  |  |  |  |  |  |
| Erdman et al. 2006 & 2007[34, 35] | 314 ♂ & 268 ♀ elite athletes, 27 sports, Canada | 88 |  | 36 |  |  | 17 |  |  |  |  |  | P-24 |  |  |  | 60 | 38 |  |
| Scofield & Unruh 2006[52] | 99 ♂ HS athletes, ~12 sports | 28 | VM-19 | 19 |  |  |  |  |  |  |  |  |  | 17 |  |  |  |  |  |
|  | 34 ♀ HS athletes, ~11 sports | 6 |  |  |  |  |  |  |  |  |  |  |  |  |  |  |  |  |  |
|  | 12 football players |  | VM-17 |  |  |  |  |  |  |  |  |  |  | 25 |  |  |  |  |  |
|  | 1 volleyball player |  | VM-0 |  |  |  |  |  |  |  |  |  |  | 0 |  |  |  |  |  |
|  | 3 basketball players |  | VM-0 |  |  |  |  |  |  |  |  |  |  | 33 |  |  |  |  |  |
|  | 7 track athletes |  | VM-29 |  |  |  |  |  |  |  |  |  |  | 14 |  |  |  |  |  |
|  | 7 soccer players |  | VM-57 |  |  |  |  |  |  |  |  |  |  | 29 |  |  |  |  |  |
|  | 5 baseball players |  | VM-40 |  |  |  |  |  |  |  |  |  |  | 40 |  |  |  |  |  |
|  | 2 softball players |  | VM-0 |  |  |  |  |  |  |  |  |  |  | 0 |  |  |  |  |  |
|  | 2 tennis players |  | VM-50 |  |  |  |  |  |  |  |  |  |  | 0 |  |  |  |  |  |
|  | 1 golfer |  | VM-0 |  |  |  |  |  |  |  |  |  |  | 0 |  |  |  |  |  |
|  | 2 X-C runners |  | VM-0 |  |  |  |  |  |  |  |  |  |  | 0 |  |  |  |  |  |
|  | 75 multisport athletes |  | VM-19 |  |  |  |  |  |  |  |  |  |  | 17 |  |  |  |  |  |
|  | 2 swimmers |  | VM-50 |  |  |  |  |  |  |  |  |  |  | 0 |  |  |  |  |  |
| Huang et al. 2006[198] | 121 ♂ Canadian athletes competing in Atlanta Oly Games (1996) | 66 | V-59  M-16 | 45 | 1 | 12 | 37 | 1 | 20 | 21 | 4 |  | P-10  A-11^p^ | 14 | 7 | 5 |  |  | Cr-4  Ca&Mg-6 |
|  | 136 ♀ Canadian athletes competing in Atlanta Oly Games (1996) | 72 | V-66  M-45 |  |  |  |  |  |  |  |  |  |  |  |  |  |  |  |  |
|  | 150 ♂ & 150 ♀ Canadian athletes competing in Sydney Oly Games (2000) | 75 | V-65  M-30 | 44 | 2 | 11 | 36 | <1 | 18 | 12 | 6 |  | P-29  A-26^p^ | 12 | 6 | 2 |  |  | Cr-4  Ca&Mg-6 |
|  | 150 ♀ Canadian athletes competing in Sydney Oly Games (2000) | 73 | V-58  M-21 |  |  |  |  |  |  |  |  |  |  |  |  |  |  |  |  |
| Striegel et al. 2006[174] | 414 ♂ & 184 ♀ master athletes (aged 50±10 yrs) | 61/64^q^ | V-35  M-35 |  |  |  |  |  |  |  |  |  | P-11 | 7 |  |  |  |  | Ω3FA-4 |
| Bent & Marsh 2006[117] | 139 college hockey players |  |  |  |  |  |  |  |  |  |  |  |  |  |  |  |  |  | ED-25  Caf-16 |
| Petroczi et al. 2007[12, 39-41] | 874 athletes, 31 sports, UK | 59 |  | 43 |  |  | 42 |  |  | 18 |  |  | P-19 | 21 | 19 | 5 |  |  | Mg-7  Caf-24 |
| Chlopicka et al. 2007[165] | 113 ♂ & 37 ♀ exercising in gyms in Poland | 81 | V-8 |  |  |  |  |  |  |  |  |  | A-8  P-4 | 20 |  |  |  |  |  |
| Duellman 2008[53]) | 61 ♂ HS football players |  |  |  |  |  |  |  |  |  |  |  | AP-28 |  |  |  |  |  |  |
| Petroczi et al. 2008[132] | 260 ♂& 133 ♀ elite British athletes, 34 sports | 48 |  | 23 |  |  | 23 |  |  | 5 |  |  | P-21 | 13 | 8 | 2 |  |  | ED-42; Caf-5 |
| Tscholl et al. 2008[66] | 736 ♂ professional soccer players in World Cup 2002 | 43 | V- 42  M-25 |  |  |  |  |  |  |  |  |  | A-8 | 9 |  |  |  |  |  |
|  | 736 ♂ professional soccer players in World Cup 2006 | 43 | V-41  M-19 |  |  |  |  |  |  |  |  |  | A-13 | 7 |  |  |  |  |  |
| Knechtle et al. 2008[108] | 20 ♂ ultra-distance runners |  | V-45  M-60 | 30 |  | 15^g^ | 20 |  | 25 | 15 | 15 | 25 |  |  |  |  |  |  | Mg-45 |
| Sekulic et al. 2008[105] | 22 ♂ Serbian sports dancers | 14 |  |  |  |  |  |  |  |  |  |  |  |  |  |  | 9 |  |  |
|  | 21 ♀ Serbian sports dancers | 24 |  |  |  |  |  |  |  |  |  |  |  |  |  |  | 24 |  |  |
| Soric et al. 2008[202] | 39 ♀ Croatian gymnasts & ballet dancers |  | V-13 |  |  |  |  |  |  |  |  |  |  |  |  |  |  |  |  |
| Tsitsimpikou et al. 2009[143] | 2,463 Doping Control & 1779 Therapeutic Use Record, Athens Oly Games (2004) | 48 | V-43 |  |  |  |  |  |  |  |  |  | AP-14 |  |  |  |  |  |  |
| Tian et al. 2009[152] | 35 ♂ & 47 ♀ collegiate athletes in Singapore, ~16 sports | 77 |  | 23 |  | 4 | 38 |  | 5 | 4 | 7 |  | AP-7 | 1 |  | 4 | 70 | 16 | Ω3FA -8;  GlCon-16;  ED-16; EC-9; BN-9 |
| Lun et al. 2009[133] | 201 ♂ & 114 ♀ elite Canadian athletes, 36 sports | 78 |  |  |  |  |  |  |  |  |  |  |  |  |  |  |  |  |  |
| Braun et al. 2009[134] | 77 ♂ & 87 ♀ elite German athletes, 26 sports | 80 | V-61  M-70 | 26 | 8 | 38^h^ | 47 |  |  | 34 | 34 |  | AP-24 | 5 |  |  | 69 | 25^i^ | Se-9; I-5  ED-25; Ω3FA-4 |
| Tscholl et al. 2010[135] | 1,947 ♂ & 1,940 ♀ track & field athletes | 66 |  |  |  |  |  |  |  |  |  |  |  |  |  |  |  |  |  |
| Knez & Peake 2010[101] | 24 ♂ triathletes | 58 |  | 53 |  |  | 59 |  | 49 | 3 | 3 | 8 | P-38  A-11^r^ | 3 | 3 |  |  |  | GL-5 |
|  | 13 ♀ triathletes | 69 |  |  |  |  |  |  |  |  |  |  |  |  |  |  |  |  |  |
| Goston 2010[17] | 526 ♂ Brazilian gym users | 45 |  | 7 |  |  |  |  |  |  |  |  | P-14  A-4^x^ | 3 |  |  | 12 |  |  |
|  | 576 ♀ Brazilian gym users | 28 |  |  |  |  |  |  |  |  |  |  |  |  |  |  |  |  |  |
| DeSilva et al. 2010[136] | 73 ♂ & 40 ♀ elite Sri Lankan athletes, ~6 sports | 94 |  | 78 |  |  |  |  | 50 | 9 | 51 |  | A-5^y^ | 27/  40^q^ |  |  | 38 |  |  |
| Dascombe 2010[18] | 36 ♂ elite Australian athletes, ~8 sports | 89 | V-32  M-28 |  |  |  |  |  |  | 32 |  |  | P-22 | 16 |  |  |  |  | GL-6; C-22 |
|  | 36 ♀ elite Australian athletes, ~8 sports | 86 | V-30  M-38 |  |  |  |  |  |  | 12 |  |  | P-6 | 2 |  |  |  |  | Caf-10 |
| Zenic et al. 2010[89] | 21 ♀ elite Croatian ballet dancers | 76 |  |  |  |  |  |  |  |  |  |  | AP-0 |  |  |  | 62 |  |  |
|  | 25 ♀ Croatian sport dancers | 18 |  |  |  |  |  |  |  |  |  |  | AP-0 |  |  |  | 18 |  |  |
|  | 23 ♀ Croatian synchronized swimmers | 53 |  |  |  |  |  |  |  |  |  |  | AP-18 |  |  |  | 29 |  |  |
| Kondric et al. 2010[124] | 50 ♂ elite table tennis players, Slovenia | 94 |  |  |  |  |  |  |  |  |  |  | P-10 |  |  |  |  |  |  |
|  | 29 ♀ elite table tennis players, Slovenian | 83 |  |  |  |  |  |  |  |  |  |  | P-7 |  |  |  |  |  |  |
| Kim et al. 2010[13] | 128 ♂ elite Korean athletes, ~14 sports | 79 |  |  |  |  |  |  |  |  |  |  |  |  |  |  |  |  |  |
|  | 100 ♀ elite athletes, Korea, ~14 sports | 82 |  |  |  |  |  |  |  |  |  |  |  |  |  |  |  |  |  |
| Nazni et al. 2010[137] | 102 ♂ elite Indian athletes, 3 sports |  |  |  |  |  |  |  |  |  |  |  |  |  |  |  | 44 | 34 |  |
| Sanchez-Oliver et al. 2011[166] | 260 ♂ using gyms in Seville, Spain |  |  |  |  |  |  |  |  |  |  |  | P-43 |  |  |  |  |  |  |
|  | 155 ♀ using gyms in Seville, Spain |  |  |  |  |  |  |  |  |  |  |  | P-3 |  |  |  |  |  |  |
| Karimian et al. 2011[77] | 250 ♂ bodybuilders in gyms in Iran | 87 | V-52  M-15 |  | 11 |  |  |  |  |  |  |  |  | 61 |  |  |  |  | Vit B_12_-19 |
|  | 250 ♀ bodybuilders in gyms in Iran | 11 | V-4  M-10 |  |  |  |  |  |  | 5 | 7 |  |  | 0 |  |  |  |  |  |
| Tsitsimpikou et al. 2011[167] | 193 ♂ athletes from gyms in Greece | 41 | V-28  M-15 |  |  |  |  |  |  |  |  |  | AP-43 | 9 |  |  | 24 |  |  |
|  | 136 ♀ athletes from gyms in Greece |  | V-10  M-13 |  |  |  |  |  |  |  |  |  | AP-2 | 0 |  |  |  |  |  |
| Bianco 2011[168] | 127♂ & 80 ♀ strength training in gyms, Italy |  |  | 6 |  |  |  |  |  |  |  |  | P-34  A-27 | 16 |  |  |  |  |  |
| Heikkinen et al. 2011[32, 33] | 261 ♂ & 185 ♀ Finnish Oly athletes, 2002 | 81 | V-67  M-37 | 54 | 0 | 18 | 28 | 1 | 3 | 21 | 11 | 7 | P-47  A-4 | 16 |  |  |  |  | Mg-16;  Ω3FA-11 |
|  | 218 ♂ & 154 ♀ Finnish Oly athletes, 2009 | 73 | V-56  M-30 | 45 | 1 | 5 | 21 | 2 | 2 | 12 | 6 | 3 | P-38  A-7 | 8 |  |  |  |  | Mg-17;  Ω3FA-19 |
| Beis et al. 2011[112] | 8 ♂ & 2 ♀ elite Ethiopian distance runners | 20 |  |  |  |  |  |  |  |  |  |  |  |  |  |  |  |  |  |
| Kim 2011[144] | 100-128 ♂ South Korean Oly athletes, 11 sports | 79 | V-56 | 56 |  |  |  |  |  |  |  |  | A-34 | 31 |  | 39^s^ |  |  |  |
|  | 82-100 ♀ South Korean Oly athletes, 11 sports | 82 | V-72 | 72 |  |  |  |  |  |  |  |  | A-15 | 10 |  | 51^s^ |  |  |  |
| Kim et al. 2011[153] | 343 ♂ South Korean collegiate athletes, ~21 sports | 43 |  | 30 |  |  | 15 |  |  |  |  |  | A-9 |  |  | 16^s^ | 5 |  |  |
|  | 136 ♀ South Korean collegiate athletes, ~21 sports | 53 |  | 30 |  |  | 21 |  |  |  |  |  | A-4 |  |  | 27^s^ | 15 |  |  |
| Russell & Pennock 2011[67] | 10 ♂ UK professional soccer players |  |  |  |  |  |  |  |  |  |  |  |  |  | 10 |  |  |  |  |
| Walsh et al. 2011[120] | 203 ♂ Irish senior schoolboy rugby players | 65 | VM-29 |  |  |  |  |  |  |  |  |  | P-44 | 29 |  |  |  |  |  |
| Dolan et al. 2011[102] | 207 ♂ triathletes | 55 |  | 40 |  |  |  |  |  |  |  |  | P-31 |  |  |  |  | 35 | Caf-20; GL-20 |
|  | 194 ♀ triathletes | 53 |  | 39 |  |  |  |  |  |  |  |  | P-26 |  |  |  |  | 22 | Caf-22; GL-22 |
| Lazic et al. 2011[23] | 657 ♂ & 255 ♀ elite Serbian athletes, 34 sports | 61 | V-53  M-22 | 43 |  |  |  |  |  |  |  |  | A-16  P-4 | 8 |  | 3 | 7 |  |  |
|  | 657 ♂ elite Serbian athletes, 34 sports | 61 |  |  |  |  |  |  |  |  |  |  |  |  |  |  |  |  |  |
|  | 255 ♀ elite Serbian athletes, 34 sports | 61 |  |  |  |  |  |  |  |  |  |  |  |  |  |  |  |  |  |
| Slattery et al. 2012[60] | 4 ♂ & 2 ♀ elite Australian swimmers | 0 |  |  |  |  |  |  |  |  |  |  |  |  |  |  |  |  |  |
| Lun 2012[19] | 163 ♂ & 277 ♀elite Canadian athletes, >10 sports | 87 |  | 16 |  |  | 4 |  |  | 3 | 2 |  | P-13^t^ |  | 2 | 3 | 24 | 11 |  |
| Diehl 2012[145] | 638 ♂ & 500 ♂ elite German Oly athletes, 51 sports | 24 |  | 6 |  |  | 11 |  | 5 | 10 | 7 | 7 | P-4 | 2 |  |  |  |  | Mg-11 |
| Rodek et al. 2012[121] | 39 ♂ & 5♀ elite Croatian sailing team members |  | VM-41 |  |  |  | 23 |  | 5 | 14 | 14 |  | AP-34 |  | 9 |  | 34 | 39 | Mg-46; PP-5  SL-7 |
| Sato et al. 2012[138] | 26 ♂ elite young (aged 13-18 yr) Japanese athletes,12 sports | 73 | V-19  M-15 |  |  |  |  |  |  |  |  |  | P-23  A-42 | 0 |  |  |  |  | UB-8 |
|  | 49 ♀ elite young (aged 13-18 yr) Japanese athletes,15 sports | 57 | V-16  M-4 |  |  |  |  |  |  |  |  |  | P-20  A-45 | 0 |  |  |  |  | UB-3 |
| Hoffman & Fogard 2012[109] | 392 ♂ & 97 ♀ ultramarathons | 76 |  | 59 |  | 5 |  | 4 | 3 | 2 | 9 |  |  |  |  |  |  |  | GlCon-35; Ω3FA-31; CoQ-5 |
| Buxton & Hagan 2012[154] | 148 ♂ & 32 ♀ university athletes, Ghana |  |  |  |  |  |  |  |  |  |  |  |  |  |  |  |  |  | ED-62 |
| Hoyte et al. 2013[155] | 276 ♂ collegiate athletes, ~10 sports | 71 |  |  |  |  |  |  |  |  |  |  |  |  |  |  |  |  | ED-84 |
|  | 186 ♀ collegiate athletes ~9 sports | 53 |  |  |  |  |  |  |  |  |  |  |  |  |  |  |  |  | ED-74 |
| Blackhouse et al. 2013[176] | 138 ♂ & 74 ♀ competitive athletes, 32 sports | 35 |  |  |  |  |  |  |  |  |  |  |  |  |  |  |  |  |  |
| Kim et al. 2013[203] | 70 ♂ & 31 ♀ Korean Judoists | 59 | V-19 |  |  |  |  |  |  |  |  |  | P-9 | 2 |  |  |  |  |  |
|  | 37 ♂ & 34 ♀ Japanese competitive judoists | 61 | V-25  M-11 |  |  |  |  |  |  |  |  |  | P-18 | 6 |  |  |  |  |  |
| Darvishi et al. 2013[156] | 173 ♂ Iranian university athletes | 45 |  | 29 |  | 5 | 19 |  | 1 | 4 | 4 |  | P-7  A-2 | 12 |  |  |  |  | Ω3FA-5 |
| Aljaloud & Ibrahim 2013[68] | 105 ♂ Saudi Arabian professional soccer players | 93 |  | 49 |  | 38 | 77 | 41 | 25 | 53 | 64 |  | A-25 | 15 |  | 27 | 83 | 55 | ED-24; Eph-12; Ω3FA-43; Caf-54; GB-10 |
| Giannopoulou et al. 2013[175] | 2,783 ♂ & ♀ Greek athletes | 38 |  |  |  |  |  |  |  |  |  |  |  |  |  |  |  |  |  |
|  | 2,013 ♂ Greek athletes |  |  |  |  |  |  |  |  |  |  |  | PA-20 |  |  |  |  |  |  |
|  | 832 ♀ Greek athletes |  |  |  |  |  |  |  |  |  |  |  | PA-11 |  |  |  |  |  |  |
| Sajber et al. 2013[61] | 55 elite Croatian swimmers | 64 |  |  |  |  |  |  |  |  |  |  |  |  |  |  |  |  |  |
| Sousa et al. 2013[14] | 200 ♂ & 92 ♀ elite Portuguese athletes,13 sports | 66 |  | 65 |  |  | 24 |  |  | 25 |  |  | P-48 | 18 |  |  | 60 |  | Mg-52; Sport Gels-25; Glu-27 |
| Wiens et al. 2014[177] | 231 ♂ & 335 ♀ young Canadian athletes, 20 sports | 98 |  | 67 |  | 32 | 66 | 48 | 26 | 27 | 43 |  | PA-72^z^ | 7 |  |  | 90 | 71 | Mg-17; ED-27; Sport Gels-9 |
| Bianco et al. 2014[169] | 434 ♂ strength training in gyms, Italy |  |  |  |  |  |  |  |  |  |  |  | P-86 |  |  |  |  |  |  |
|  | 127 ♀ strength training in gyms, Italy |  |  |  |  |  |  |  |  |  |  |  | P-21 |  |  |  |  |  |  |
| Sekulic et al. 2014[204] | 105 ♂ elite Croatian rugby union players | 52 |  |  |  |  |  |  |  |  |  |  |  |  |  |  |  |  |  |

*As reported in article; ^a^Where a specific supplement was not discussed in a study, the space in the prevalence table is blank. Where authors specifically stated that no athletes used a particular dietary supplement, the incidence is listed as “0”. Where at least 4% of the sample used a particular dietary supplement and the dietary supplement was not listed in the table columns, the dietary supplement is reported in the last table column. Where possible, the data are separated by sex and sport unless the authors did not separate the data in this manner and then the data were expressed for the entire group (e.g., men and women combined, all sports combined). If no nationality is reported, the sample was from the United States. ^b^Combines amino acid & protein categories ^c^Combines protein powders & amino acid powders; ^d^Combines carbohydrate & electrolyte drinks; ^f^Study provided only ranges, dependent on sport & does not break down data by individual sport; ^g^Combines B complex & folic acid; ^h^Combines Vitamins B_1_, B_6_, B_9_ & B_12_ & is likely an overestimate; ^i^Combines protein bars & carbohydrate bars; ^m^Excludes sport drinks; ^n^Only vitamin & protein supplements; ^o^Combines amino acids, glutamine, & arginine; ^p^Combines amino acid & glutamine; ^q^Different parts of paper report different values; ^r^Combines branched chain amino acids & other amino acids; ^s^Combines Korean ginseng & Korean red ginseng; ^t^Combines protein powders & protein bars; ^u^Tables only report 1,066 athletes; ^v^Combines amino acids & glutamine; ^w^Combines glutamine, arginine, ornithine, & other amino acids, ^X^Combines branched-chain amino acids & other amino acids; ^y^Only branched-chain amino acids; ^z^Combines branched-chain amino acids, beta-alanine, glutamine, and protein powders

Abbreviations/Symbols: DS=dietary supplement, Suppl=Supplement, yr=year, ♂=men, ♀=women, Vit or V=vitamin, minl or M=mineral, VM=vitamins or minerals, MultiVit=multivitamin, w=with, wo=without, AA or A=amino acid, Prot or P=protein, cmpx=complex, Fe=iron, Ca=calcium, Zn=zinc, K=potassium, Mg=magnesium, Se=selenium, I=iodine, Cr=Chromium, Cu=copper, Caf=caffeine, Car=carnitine, Glu=glutamine; GL=glucosamine, GlCon=glucosamine/chondroitin, CoQ=coenzyme Q10, Ω3FA=omega-3-fatty acids/fish oil/cod liver oil, GB=*ginkgo biloba*, ED=energy drinks, UB=ubiquinone, BY=brewer’s yeast, DL=desiccated liver, PE=papaya enzymes, BP=bee pollen, Dol=dolomite, HR=horseradish, Gar=garlic, WG=wheat germ, GS=goldenseal, Eph=ephedra, Cham=chamomile, EC=essence of chicken, BN=bird’s nest, GT=green tea, KK=kava kava, CP=chromium picolinate, PP=propolis; Car=carnitine; UK=United Kingdom, US=United States, HS=high school, Oly=Olympic, X-C=cross country
